# Longitudinal Structural MRI Findings in Individuals at Genetic and Clinical High Risk for Psychosis: A Systematic Review

**DOI:** 10.3389/fpsyt.2021.620401

**Published:** 2021-02-02

**Authors:** Kate Merritt, Pedro Luque Laguna, Ayela Irfan, Anthony S. David

**Affiliations:** ^1^Division of Psychiatry, Institute of Mental Health, University College London, London, United Kingdom; ^2^The Cardiff University Brain Research Imaging Centre (CUBRIC), Cardiff University, Cardiff, United Kingdom

**Keywords:** high risk psychosis, MRI, DTI, neuroimaging, clinical high risk (CHR), ultra high risk (UHR), psychotic like experiences, genetic high risk for psychosis

## Abstract

**Background:** Several cross-sectional studies report brain structure differences between healthy volunteers and subjects at genetic or clinical high risk of developing schizophrenia. However, longitudinal studies are important to determine whether altered trajectories of brain development precede psychosis onset.

**Methods:** We conducted a systematic review to determine if brain trajectories differ between (i) those with psychotic experiences (PE), genetic (GHR) or clinical high risk (CHR), compared to healthy volunteers, and (ii) those who transition to psychosis compared to those who do not.

**Results:** Thirty-eight studies measured gray matter and 18 studies measured white matter in 2,473 high risk subjects and 990 healthy volunteers. GHR, CHR, and PE subjects show an accelerated decline in gray matter primarily in temporal, and also frontal, cingulate and parietal cortex. In those who remain symptomatic or transition to psychosis, gray matter loss is more pronounced in these brain regions. White matter volume and fractional anisotropy, which typically increase until early adulthood, did not change or reduced in high risk subjects in the cingulum, thalamic radiation, cerebellum, retrolenticular part of internal capsule, and hippocampal–thalamic tracts. In those who transitioned, white matter volume and fractional anisotropy reduced over time in the inferior and superior fronto-occipital fasciculus, corpus callosum, anterior limb of the internal capsule, superior corona radiate, and calcarine cortex.

**Conclusion:** High risk subjects show deficits in white matter maturation and an accelerated decline in gray matter. Gray matter loss is more pronounced in those who transition to psychosis, but may normalize by early adulthood in remitters.

## Introduction

Schizophrenia is posited to be a neurodevelopmental disorder, in which genetic and environmental factors interplay (1). A better understanding of the neurobiological changes that occur prior to the onset of disorder could identify new treatment targets to prevent transition to schizophrenia. With this aim, numerous studies have reported cross-sectional differences in brain structure between subjects at high risk of developing schizophrenia and healthy volunteers. Such studies typically involve young persons ranging from adolescence to early adulthood, at a time of dynamic brain maturation. In order to fully recognize how brain development is altered in the run up to schizophrenia, it is key to conduct longitudinal studies in the prodrome. Therefore, we aimed to summarize longitudinal studies that track neuroimaging measures over time in individuals at genetic or clinical high risk of developing schizophrenia, and those in the general population who experience psychotic symptoms.

In general, neuroimaging studies examine three types of high risk cohorts; (i) help-seeking individuals who exhibit a reduction in functioning as well as attenuated psychotic symptoms or genetic risk for schizophrenia (clinical high risk; CHR, also known as an ultra-high risk of developing psychosis; UHR, or at-risk mental state; ARMS), (ii) individuals who exhibit attenuated psychotic symptoms and are not help seeking (psychotic experiences group; PE), and (iii) those with at least one relative diagnosed with schizophrenia (genetic high risk group; GHR) (2). The highest rate of transition is seen in the CHR group (36% after 3 years) (3), whereas transition rates are lower in the GHR (10–15%) (4) and PE groups (10%) (5). A meta-analysis of 14 cross-sectional voxel-based morphometry studies reported reduced gray matter in the anterior cingulate, middle frontal and temporal cortex in UHR subjects compared to healthy volunteers, and gray matter reductions were [more pronounced in schizophrenia] (6). A recent meta-analysis also reported decreased gray matter in frontal brain regions but increased gray matter volume in cingulate, right thalamus, left superior temporal gyrus and right fusiform gyrus (7), and it has been proposed that consequent volume reductions may occur with psychosis onset (8). In normal development, gray matter volume falls relative to total brain volume during adolescence and plateaus in the twenties (9). It is unclear from cross-sectional studies whether high risk subjects have a stable trait of lower gray matter, whether these differences emerge during brain maturation, or emerge following the onset of psychosis.

White matter development is more extended over the lifespan relative to gray matter. White matter volume increases over childhood and adolescence to reach peak levels in mid-adulthood, which coincides with the average age of schizophrenia onset. During this time the connectome is strengthened by increasing axonal diameter and myelination of white matter tracts (10). Fractional anisotropy (FA) values from diffusion tensor imaging (DTI) are sensitive to these changes in white matter microstructure. Cross-sectional studies report reduced FA in subjects at genetic and clinical high risk compared to healthy volunteers (11). As FA typically increases until mid-adulthood, it is not clear whether high risk groups have altered white matter trajectories or a stable deficit over time, or whether they recover from this deficit.

Longitudinal studies can also determine whether brain trajectories differ in high risk subjects who go on to transition to schizophrenia, compared to those who do not. A number of clinical services are now specialized to identify CHR individuals, however two-thirds of CHR individuals do not go on to develop psychosis, and it is not currently possible to predict who will transition (3). A meta-analysis of 25 cross-sectional studies found decreased prefrontal, cingulate, insular and cerebellar gray matter volume in subjects who went on to transition to psychosis compared to those who did not (12). Moreover, a large multisite study found less parahippocampal gray matter volume in those who later developed psychosis (13). Machine learning of brain structure measures alone seem to be insufficient for individual outcome prediction, although the addition of clinical measures increases prediction accuracy (14). Longitudinal measures may offer more sensitivity to detect differences between transition groups, partly by taking into account individual differences but also by revealing trajectories which may aid better prediction models.

In this systematic review, we included longitudinal structural and diffusion weighted Magnetic Resonance Imaging (MRI) studies examining participants at an enhanced risk of developing schizophrenia. We aimed to examine whether neurodevelopmental trajectories differ between (i) those with either psychotic experiences (PE), genetic (GHR) or clinical high risk (CHR, also known as UHR), compared to healthy volunteers, and (ii) those who transition compared to those who do not transition to psychosis. We hypothesized, first, that altered neurodevelopmental trajectories would be present in high risk populations (PE, GHR, and CHR) in comparison to healthy volunteers. Secondly, we predicted that these patterns of altered brain development would be more pronounced in subjects who remain symptomatic or transition to psychosis, compared to those who remit or do not transition.

## Methods

The systematic review was pre-registered on PROSPERO (CRD42020199065), and adhered to PRISMA guidelines. PubMed (including MEDLINE) and PsychInfo databases were searched to identify journal articles published from inception until 6 June 2020, to search titles and abstracts using the following search terms: (mri OR fmri OR dti OR brain OR “grey matter” OR “gray matter” OR “white matter” OR neuroimaging OR fa OR “fractional anisotropy” OR myelin OR magnetic resonance imaging OR diffusion tensor imaging OR “resting state” OR structural OR connectivity OR “diffusion weighted” OR “DW MRI” OR “Brain development”) AND (“Psychosis risk” OR UHR OR CHR OR “clinical High Risk” OR “at risk mental state” or GHR or “psychotic experience” or “psychosis spectrum” or “clinical risk” OR [“genetic risk” and (psychosis or schizophrenia)] OR [“ultra high risk” and (psychosis or schizophrenia)] OR [“ultra-high risk” and (psychosis or schizophrenia)] OR [“high risk” and (psychosis or schizophrenia)] OR “risk” and (psychosis or schizophrenia) OR (emerging and psychosis) OR (psychopathology and youth) OR (prodromal and psychosis) OR (subthreshold and psychosis)) AND (follow^*^ OR longitudinal^*^ OR prospective OR “birth cohort” or development or trajectory OR progressive). References of previous reviews were also searched. All longitudinal studies reporting structural MRI measures in a high risk group with a comparison group (either healthy volunteers, or comparisons between transition and non-transition high risk groups), or exploring an association with psychotic symptom scores or polygenic risk score for schizophrenia were included in the systematic review. Studies using overlapping samples were included, providing different MRI analyses had been applied. Study quality was assessed by the following criteria: (i) blinded groups at the analysis stage, (ii) participant drop out reported, and (iii) correction for multiple comparisons.

Clinical high risk subjects, also defined as ultra high risk or individuals meeting an at risk mental state, are referred to as “CHR” in this review. CHR, GHR, and PE subjects are referred to collectively as high risk subjects (HR).

## Results

**Figure 1:**
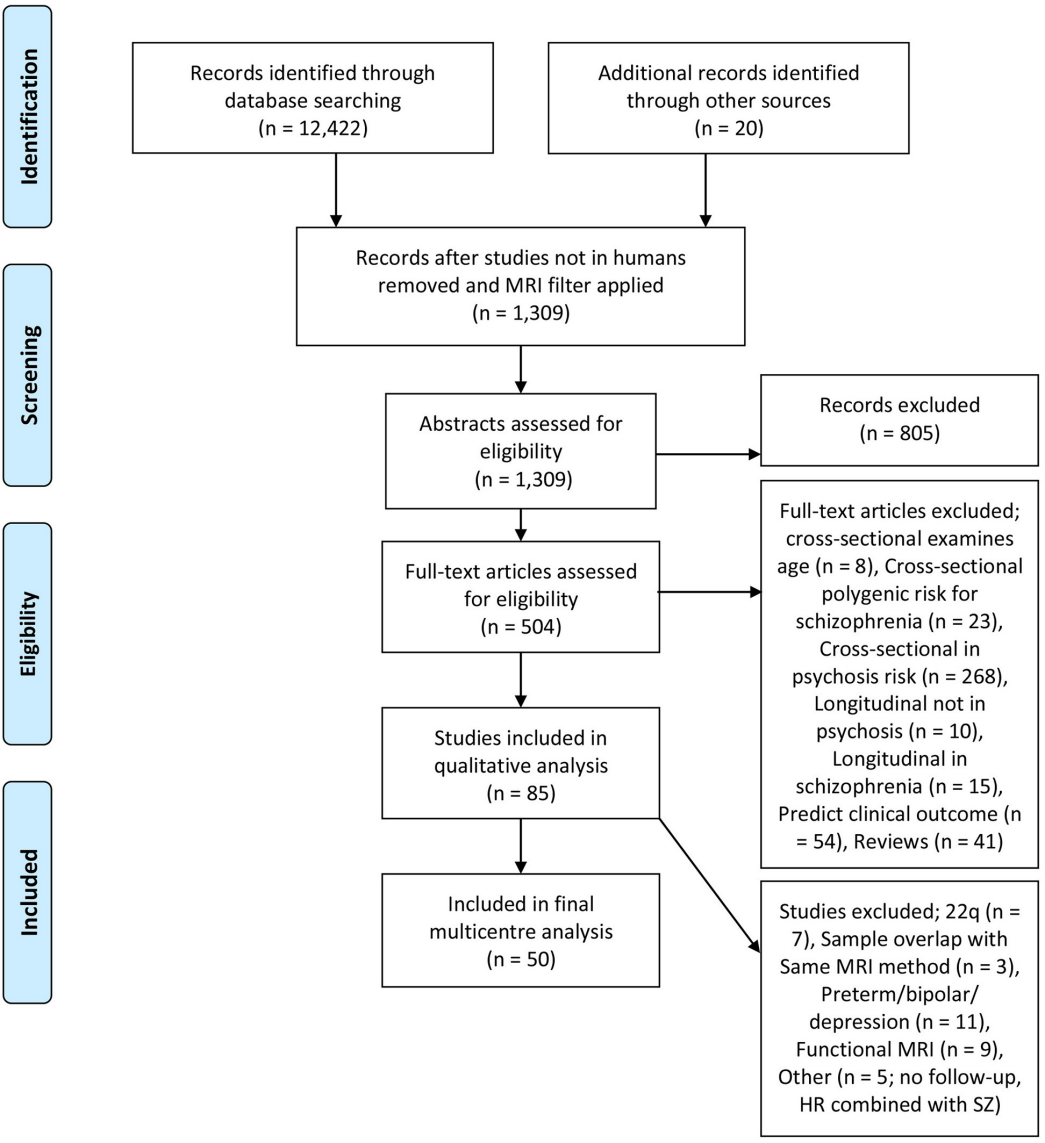
PRISMA diagram.

The literature search identified 50 studies from 22 high risk (HR) cohorts, including 2,473 HR subjects and 990 healthy volunteers (HV) (Figure 1, PRISMA diagram). Twenty-six studies examined CHR subjects (also defined as ultra high risk or individuals meeting an at risk mental state), 19 studies examined GHR subjects, and 5 studies examined individuals with PE. Thirty-eight studies examined gray matter (Table 1) and 18 examined white matter (Table 2). The average age of the HR group was 21.4 years (SD 9.7) at baseline, with the follow-up scan occurring on average 3.4 years later (SD 2.7). CHR groups were assessed by the Comprehensive Assessment of At Risk Mental State, the Structured Interview for Prodromal Syndromes, the Basel Screening Instrument for Psychosis, and the Bonn Scale for the Assessment of Basic Symptoms. Four cohorts assess PE; (1) The Edinburgh Study of Comorbidity examines adolescents with cognitive impairment and schizotypal features which have been classified as PE in this review, assessed via the Structured Interview for Schizotypy, (2) The IMAGEN study used the Community Assessment of Psychic Experiences (CAPE) which takes into account frequency and distress of symptoms, (3) Calvo et al., used the SOCRATES template, which documents the presence of perceptual abnormalities and unusual thought content with no frequency or duration criteria, (4) The PNC cohort used the Scale of Prodromal Symptoms, and individuals were required to possess significant sub-threshold symptoms that persisted for at least two clinical assessments (on average 20 months apart). Detailed summaries of each study can be found in the Supplementary Tables 1, 2.

**Table 1:** Studies of gray matter indices included in the systematic review, with summary statistics on the final row.

**Cohort**	**References**	**CHR = 1, GHR = 2, PE = 3**	**HV (*n*)**	**HR (*n*)**	**HR-T (*n*)**	**HR-NT (*n*)**	**HR-S (*n*)**	**HR-NS (*n*)**	**Age of HR (yrs)**	**Follow up (yrs)**	**AP naive**	**Cortical thick-ness**	**Volume**	**Surface area**	**Quality: B/DO/CMC**
Toho University	Katagiri et al. (15)	1	16	42	5	37			22.3	1	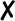		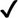		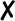 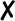 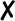
ADAPT	Damme et al. (16)	1	38	81					19	1	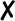			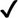	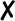 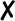 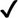
NAPLS	Cannon et al. (17)	1	135	274	35	239			19.3	1	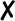	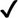			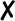 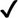 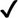
	Chung et al. (18)	1	135	274	35	239			19.3	1	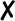	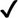			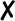 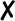 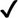
	Chung et al. (19)	1	132	267	37	230			19.3	2	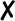	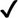			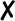 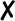 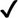
Singapore	Ho et al. (20)	1	54	93			41	52	21.4	2	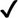		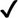		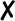 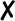 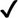
NIMH COS Study	Gogtay et al. (21)	2	52	52					16.2	10	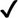	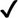			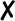 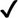 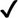
	Mattai et al. (22)	2	86	43					13.4	6	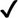	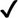			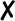 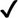 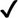
	Zalesky et al. (23)	2	102	86					18	10	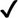	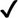			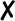 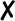 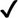
	Greenstein et al. (24)	2	110	80					14.9	10	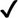		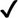		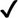 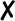 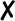
	Mattai et al. (25)	2	79	78					14.9	10	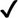		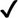		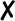 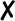 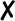
Sibling pair, Utrecht	Brans et al. (26)	2	33	11					41.2	5	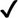		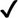		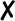 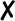 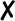
Twin pair, Utrecht	Hedman et al. (27)	2	54	19					38.7	5	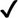	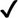		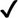	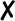 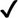 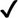
Dutch Prediction of Psychosis	Ziermans et al. (28)	1	30	43	8	35			16.1	2	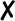	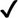	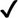		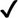 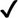 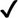
	de Wit et al. (29)	1	24	35			18	17	15.4	6	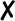	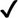	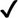	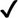	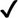 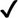 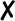
Edinburgh High Risk Study	Lawrie et al. (30)	2	20	66			19	47	23.1	2	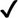		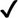		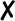 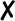 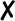
	Job et al. (31)	2	19	65			18	47	21.4	2	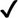		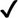		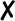 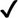 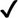
	Mcintosh et al. (32)	2	36	146	17	72	57		21.2	10	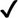		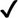		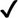 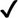 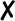
	Bois et al. (33)	2	36	142	17	68	57		21	2	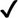	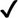		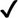	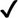 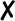 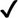
	Bois et al. (34)	2	36	142	17	68	57		21	2	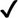		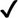		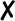 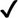 -
Edinburgh Study of Comorbidity	Moorhead et al. (35)	3	45	53					15.9	1.5	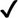		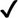		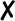 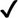 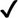
	McKechanie et al. (36)	3	NA	43					16.2	6	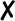		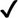		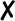 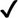 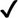
FEPSY project	Walter et al. (37)	1	NA	23	10	13			24.2	5	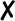		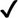		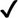 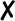 -
	Walter et al. (38)	1	NA	18	8	10			25.7	5	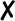		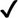		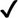 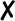 -
	Borgwardt et al. (39)	1	NA	20	10	10			24.7	4	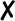		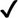		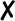 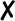 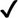
OASIS	Fusar-Poli et al. (40)	1	14	22	5	17			24.5	2	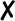		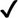		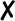 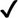 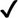
PACE	Pantelis et al. (41)	1	NA	21	10	11			19.7	3	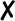		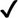		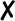 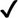 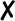
	Sun et al. (42)	1	NA	35	12	23			19.9	4	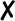			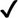	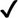 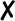 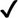
	Takahashi et al. (43)	1	22	35	12	23			19.9	4	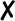		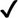		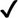 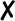 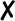
	Takahashi et al. (44)	1	20	31	11	20			19.9	4	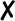		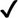		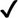 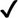 -
Ludwig Maximilians	Koutsouleris et al. (45)	1	28	25	12	13			23.2	4	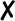		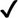		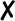 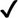 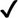
Western Psychiatric Institute and Clinic	Bhojraj et al. (46)	2	36	56					15.4	1	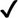	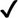		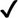	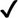 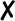 -
	Bhojraj et al. (47)	2	27	23					15.4	1	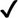		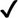		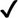 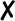 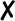
	Prasad et al. (48)	2	33	31					16.3	1	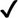	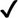	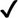	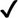	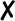 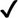 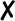
IMAGEN	Yu et al. (49)	3	NA	706					14.4	4	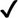		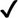		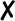 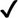 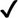
Dublin & Kildare	Calvo et al. (50)	3	25	25					13.5	2	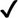		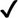		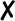 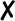 -
Barcelona	Sugranyes et al. (51)	2	34	58					11.3	2.3	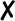	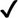		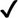	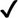 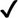 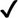
	Sugranyes et al. (52)	2	49	79			20	59	12.1	4	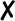	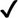	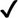	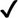	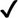 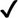 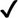
Totals:		17 GHR, 17 CHR, 4 PE	1560	3343	261	1128	287	222	Mean 19.7	Mean 3.9	19	14	26	9	Mean 1.4/3

*GHR, Genetic high risk; CHR, Clinical high risk; T, transition; NT, no transition; S, symptomatic; NS, no symptoms; HR, high risk; HV, Healthy Volunteers; PE, psychotic experiences; AP, antipsychotic medication; NA, Not Applicable; Quality, Blind/Drop-Out Reported/Correct for Multiple Comparisons*.

**Table 2:** Studies of white matter indices included in the systematic review, with summary statistics on the bottom row.

**Cohort**	**References**	**CHR = 1, GHR = 2, PE = 3**	**HV (*n*)**	**HR (*n*)**	**HR-T (*n*)**	**HR-NT (*n*)**	**Age of HR (yrs)**	**Follow up (yrs)**	**AP naive**	**MRI analysis**	**Quality: blind/drop-out/correct for multiple comparisons**
Sibling pair cohort	Brans et al. (26)	2	33	11			41.2	5	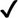	Struct: Volume	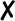 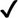 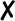
Dutch Prediction of Psychosis	Ziermans et al. (28)	1	30	43	8	35	16.1	2	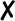	Struct: Semiautomated volume + VBM	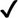 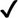 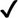
	de Wit et al. (29)	1	24	35	18	17	15.4	6	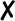	Struct: Volume	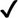 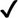 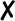
Edinburgh Study of Comorbidity	Moorhead et al. (35)	3	45	53			15.9	1.5	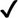	Struct: TBM	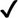 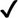 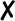
Ludwig-Maximilians	Koutsouleris et al. (45)	1	28	25	12	13	23.2	4	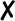	Struct: VBM Multivariate	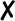 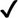 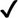
PACE	Walterfang et al. (53)	1	NA	21	10	11	20.5	1.3	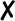	Struct: VBM	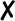 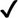 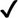
NIMH COS study	Gogtay et al. (54)	2	57	49			16.1	5	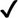	Struct: TBM	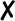 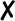 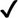
LBC1936 birth cohort	Alloza et al. (55)	NA	NA	488			76.4	3	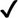	DTI: Tractometry and connectomic	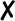 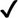 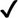
Toho University	Katagiri et al. (56)	1	16	42	5	37	22.3	1	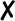	Struct: ROI volume (Corpus Callosum)	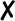 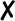 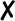
	Saito et al. (57)	1	16	46	7	39	22.9	1	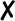	DTI: Tractometry (Corpus Callosum)	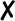 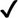 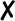
	Katagiri et al. (58)	1	16	41	7	34	22.8	1	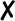	DTI: cross-sectional whole-brain TBSS followed by longitudinal ROI analysis	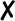 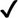 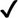
Copenhagen	Krakauer et al. (59)	1	23	30			24.1	1	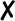	DTI: Whole-brain TBSS	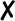 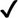 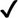
ADAPT	Bernard et al. (60)	1	21	26			18.7	1	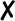	DTI: Tractometry (hippocampal–thalamic)	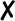 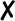 -
	Mittal et al. (61)	1	15	15			18.5	1	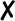	DTI: Atlas-based TBSS ROI analysis (Superior Cerebella Peduncles)	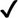 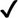 -
	Bernard et al. (62)	1	24	26			18.7	1	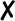	DTI: Tractometry (Cerebello-thalamo-cortical)	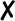 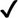 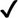
Genetic Risk and Outcome of Psychosis	Domen et al. (63)	2	49	55			30.9	3	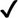	DTI: Atlas-based TBSS ROI analysis (2x19 regions)	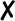 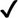 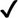
OASIS	Carletti et al. (64)	1	32	22	5	17	23.4	2	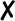	DTI: Cluster-level suprathreshold voxel analysis	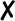 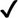 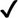
PNC	Roalf et al. (65)	3	89	38			15.5	2	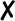	DTI: Whole-brain TBSS followed by Atlas-based ROI analysis	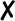 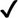 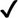
Totals:		3 GHR, 12 CHR, 2 PE, 1 Older adults	518	1,066	72	203	Mean 24.6	Mean 2.3	5	8 Structural, 3 Tractometry, 1 Connectomic, 2 Whole-brain-TBSS, 3 ROI-TBSS, 1 Cluster-level DTI	Mean 1.7/3

*GHR, Genetic high risk; CHR, Clinical high risk; T, transition; NT, no transition; S, symptomatic; NS, no symptoms; HR, high risk; HV, Healthy Volunteers; PE, psychotic experiences; AP, antipsychotic medication; NA, Not Applicable*.

### Longitudinal Brain Trajectories in High Risk Subjects Compared to Healthy Volunteers

For studies of gray matter comparing HR groups to healthy volunteers (HV), 20 out of 24 studies measuring brain volume, cortical thickness, or surface morphology, reported altered gray matter trajectories in HR (Table 3). Altered gray matter development is seen regardless of the type of HR group examined; a significant change over time compared to HV was reported in 13 out of 16 studies of GHR, 4 out of 5 studies in CHR, and 3 out of 3 studies of PE. Significant results were primarily in the temporal (16 studies), frontal (9 studies), parietal lobe (6 studies), cingulate cortex (6 studies), and whole brain (6 studies).

**Table 3:** List of studies examining gray matter indices, indicating significant differences between groups either at baseline (BL) or a significant group x time interaction.

**Dataset**	**References**	**BL: HR vs. HV**	**Time:** ** HR vs. HV**	**Time: CHR-T vs. CHR-NT**	**BL:** ** Predict Transition**	**Significant brain regions for HR vs. HV over time**											**Significant brain regions for CHR-T vs. CHR-NT over time**									
						**Whole brain**	**Frontal**	**ACC**	**Temporal**	**Parietal**	**Occipital**	**Thalamus**	**Amygdala**	**Basal Ganglia**	**Cerebellum**	**Ventricle**	**Whole brain**	**Frontal**	**Insula**	**ACC**	**Temporal**	**Parietal**	**Occipital**	**Nucleus Accumbens**	**Cerebellum**	**Ventricle**
Toho University	Katagiri et al. (15)	NA	NA	NA	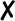																					
ADAPT	Damme et al. (16)	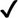	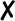	NA	NA																					
NAPLS	Cannon et al. (17)	NA	NA	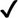	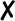													↓ CT								↑ Vol
	Chung et al. (18)	NA	NA	NA	NA																					
	Chung et al. (19)	NA	NA	NA	NA																					
Singapore	Ho et al. (20)	NA	NA	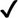	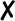																↓ Vol					
NIMH COS Study	Gogtay et al. (21)	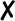	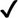	NA	NA		↓ CT		↓ CT	↓ CT																
	Mattai et al. (22)	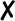	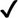	NA	NA		↓ CT		↓ CT	↓ CT																
	Zalesky et al. (23)	NA	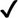	NA	NA				↓ Con		↓ Con															
	Greenstein et al. (24)	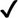	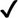	NA	NA										↓ Vol											
	Mattai (25)	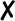	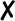	NA	NA																					
Sibling pair, Utrecht	Brans et al. (26)	NA	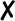	NA	NA																					
Twin pair, Utrecht	Hedman et al. (27)	NA	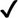	NA	NA	↓ CT			↓ CT																	
Dutch Prediction Psychosis	Ziermans et al. (28)	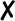	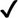	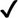	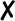				↓ CT								↓ Vol			↓ CT	↓ CT	↓ CT	↓ CT			
	de Wit et al. (29)	NA	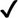	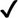	NA	↔ SA	↔ SA ↑ Vol	↓ SA	↓ Vol ↓ CT	↔ SA ↓ GI	↓ SA ↓ GI ↓ Vol	↓ Vol				↑ Vol	↓ CT^*^ SA^*^	↓ Vol^*^↓ CT^*^ ↓ SA		↓ Vol ↑ GI	↓ Vol^*^↓ CT^*^↔ CT	↓ Vol^*^↓ CT^*^		↓ Vol^*^		↑ Vol
Edinburgh High Risk Study	Lawrie et al. (30)	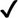	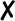	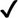	NA																↓ Vol					
	Job et al. (31)	NA	Effect of time	Effect of time	NA			↓ Vol	↓ Vol	↓ Vol					↓ Vol											
	Mcintosh et al. (32)	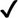	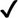	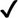	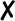	↓ Vol	↓ Vol		↓ Vol									↓ Vol								
	Bois et al. (33)	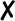	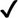	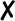	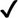	↓ CT, ↔ SA	↓ CT	↓ CT			↔ CT															
	Bois et al. (34)	NA	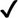	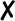	NA				↔ Vol																	
Edinburgh Study of Comorbidity	Moorhead et al. (35)	NA	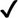	NA	NA				↓ Vol				↓ Vol													
	McKechanie et al. (36)	NA	NA	NA	NA																					
FEPSY project	Walter et al. (37)	NA	NA	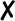	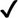																					
	Walter et al. (38)	NA	NA	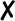	NA																					
	Borgwardt et al. (39)	NA	NA	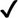	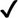													↓ Vol			↓ Vol	↓ Vol			↓ Vol	
OASIS	Fusar-Poli et al. (40)	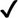	Effect of time	Effect of time	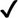		↑↓ Vol	↑ Vol						↓ Vol	↑ Vol						↓ Vol					
PACE	Pantelis et al. (41)	NA	NA	Effect of time	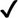													↓ Vol		↓ Vol	↓ Vol		↑ Vol		↓ Vol	
	Sun et al. (42)	NA	NA	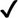	NA													↓ SA								
	Takahashi et al. (43)	NA	NA	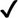	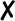																↓ Vol					
	Takahashi et al. (44)	NA	NA	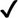	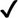														↓ Vol							
Ludwig Maximilians	Koutsouleris et al. (45)	NA	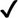	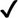	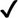		↓ Vol	↓ Vol	↓ Vol	↓ Vol	↓ Vol			↓ Vol	↓ Vol	↑ Vol				↓ Vol		↓ Vol				↑ Vol
Western Psychiatric Institute and Clinic	Bhojraj et al. (46)	NA	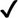	NA	NA				↓ SA																	
	Bhojraj et al. (47)	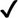	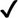	NA	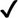		↓ Vol	↓ Vol	↓ Vol																	
	Prasad et al. (48)	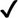	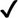	NA	NA	↓ SA	↓ SA, ↓ Vol, ↑ CT		↓ Vol, ↑ CT	↓ Vol, ↑ CT	↓ SA, ↓ Vol															
IMAGEN	Yu et al. (49)	NA	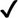	NA	NA				↔ Vol																	
Dublin & Kildare	Calvo et al. (50)	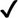	Effect of group	NA	NA				↓ Vol^*^																	
Barcelona	Sugranyes et al. (51)	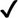	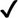	NA	NA	↔ SA																				
	Sugranyes et al. (52)	NA	NA	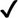	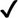												↓ CT ↓ SA^*^ NT vs. HV: ↔ SA+Vol						↓ CT			
TOTAL:		9/14	20/24	15/19	9/15	6	9	6	16	6	4	1	1	2	4	2	3	6	1	4	9	4	3	1	3	3
		BL: HR vs. HV	Time: HR vs. HV	Time: CHR-T vs. CHR-NT	BL: Predict Transition	Whole brain	Frontal	ACC	Temporal	Parietal	Occipital	Thalamus	Amygdala	Basal Ganglia	Cerebellum	Ventricle	Whole brain	Frontal	Insula	ACC	Temporal	Parietal	Occipital	Nucleus Accumbens	Cerebellum	Ventricle

*Arrows indicate altered trajectories between groups in specified brain region, ↓ indicate a decrease in the HR or CHR-T group compared to HV or CHR-NT respectively, ↑ indicate an increase and ↔ indicate no change over time in HR whereas an increase/decrease is seen in HV. HR, high risk; HV, healthy volunteer; CHR-T, clinical high risk transition; CHR-NT, no transition; ACC, Anterior cingulate cortex; NA, Not Applicable (for example studies examine other contrasts such as medicated vs. unmedicated); CT, cortical thickness; Vol, gray matter volume; Conn, structural connectivity; SA, surface area; GI, gyrification*.

* *volume was lower in HR vs. HV at both timepoints but there was no group × time interaction*.

In the temporal cortex, the majority of studies (13 studies) found a reduction in cortical thickness or volume over time in HR subjects, whereas 2 studies reported no change over time in HR, but a decrease (48) or an increase in HV (34). One study did not detect changes over time but instead found lower hippocampal volume at both timepoints in HR (50). In the frontal cortex and cingulate, gray matter volume, cortical thickness and surface morphology reduced over time in HR in the majority of studies, with the exception of two studies. One study found increased gray matter in the right inferior frontal gyrus and anterior cingulate, but reduced in bilateral superior frontal gyrus, although an interaction between group and time was not assessed (40). One study found less steep decreases in surface area over time in CHR in frontal and parietal areas (29). In the parietal lobe, the majority of studies reported reduced gray matter volume and cortical thickness over time in HR, bar the aforementioned study (29) and another study which found increased cortical thickness but reduced volume over time (48). For whole brain, differences were found in cortical surface area and gray matter. A number of studies found smaller longitudinal decreases in global surface area in HR compared to healthy volunteers (29, 33, 51), although one study found a reduction in GHR (48). For whole brain gray matter, greater reductions over time in GHR compared to HV were reported (32), and more pronounced global cortical thinning in discordant twin pairs compared with healthy control twin pairs, although no difference in surface area was observed in this study (27).

For studies of white matter comparing HR groups to HV, 9 out of 13 studies reported altered trajectories in HR groups (5/7 studies in CHR, 2/3 studies in GHR, 1/2 in PE, 1 in subjects with polygenic risk scores for schizophrenia). Altered trajectories were reported principally in whole brain, in the cingulum and in the thalamic radiation (3 studies) amongst other regions (Table 4). Studies measuring white matter volume found reduced volume over time or showed smaller increases compared to HV, in the whole brain, parietal lobes and in the corpus callosum of HR subjects (3 out of 5 volume studies). Slower white matter growth in the parietal lobe normalized by age 14 in GHR subjects (54).

**Table 4:** List of studies examining white matter, ticks indicate significant differences between groups either at baseline (BL) or a significant group × time interaction.

**Dataset**	**References**	**BL: HR vs. HV**	**Time: HR vs. HV**	**Time: CHR-T vs. CHR-NT**	**BL: Predict Transition**	**Significant brain regions for HR vs. HV over time**																**Significant brain regions for CHR-T!!break vs. CHR-NT over time**						
						**Whole brain**	**Cingulum**	**Fronto-occipital fasciculus**	**Cerebellum**	**Cerebello-thalamo-cortical**	**Parietal**	**Splenium**	**Arcuate**	**Thalamic radiations**	**SLF**	**Inferior longitudinal fasciculus**	**Thalamic – hippocampal**	**Internal capsule**	**Forceps major**	**Corticospinal**	**Corpus collosum**	**Whole brain**	**Fronto-occipital fasciculus**	**Cerebellum**	**Calcarine cortex**	**Internal capsule**	**Corona radiata**	**Corpus collosum**
Sibling pair cohort	Brans et al. (26)	NA	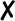	NA	NA																							
Dutch Prediction Psychosis	Ziermans et al. (28)	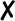	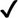	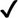	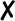	↔ Vol																↓ Vol						
	De Wit et al. (29)	NA	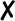	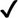	NA																							↓ Vol^*^
Edinburgh Study Co-morbidity	Moorhead et al. (35)	NA	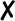	NA	NA																							
Ludwig-Maximilians	Koutsouleris et al. (45)	NA	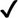	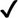	NA																↓ Vol							↓ Vol
PACE	Walterfang et al. (53)	NA	NA	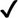	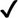																		↓ Vol	↑ Vol	↓ Vol			
NIMH COS study	Gogtay et al. (54)	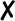	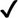	NA	NA						↔ Vol																	
LBC1936 birth cohort	Alloza et al. (55)	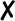	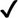	NA	NA		↑ MD					↑ MD	↑ MD	↑ MD														
Toho University	Katagiri et al. (56)	NA	NA	NA	NA																							
	Saito et al. (57)	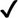	NA	NA	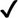																							
	Katagiri et al. (58)	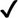	NA	NA	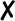																							
Copenhagen	Krakauer et al. (59)	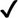	Effect of time	NA	NA										↑ FA													
ADAPT	Bernard et al. (60)	NA	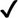	NA	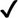												↓↔ FA											
	Mittal et al. (61)	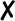	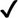	NA	NA				↓ FA																			
	Bernard et al. (62)	NA	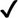	NA	NA					↓ FA																		
Genetic Risk and Outcome of Psychosis	Domen et al. (63)	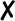	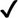	NA	NA	↓ FA	↓ FA							↓ FA				↔ FA										
OASIS	Carletti et al. (64)	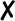	NA	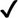	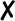																		↓ FA			↓ FA	↓ FA	↓ FA
PNC	Roalf et al. (65)	NA	Effect of group	NA	NA	↓ FA^*^	↓ FA^*^	↓ FA^*^						↓ FA^*^	↓ FA^*^	↓ FA^*^			↓ FA^*^	↓ FA^*^								
TOTAL:		3/9	9/13	5/5	3/6	3	3	1	1	1	1	1	1	3	2	1	1	1	1	1	1	1	2	1	1	1	1	3
		BL: HR vs. HV	Time: HR vs. HV	Time: CHR-T vs. CHR-NT	BL: Predict Transition	Whole brain	Cingulum	Fronto-occipital fasciculus	Cerebellum	Cerebello-thalamo-cortical	Parietal	Splenium	Arcuate	Thalamic radiations	SLF	Inferior longitudinal fasciculus	Thalamic–ippocampal	Internal capsule	Forceps major	Corticospinal	Corpus collosum	Whole brain	Fronto-occipital fasciculus	Cerebellum	Calcarine cortex	Internal capsule	Corona radiata	Corpus collosum

Arrows indicate significant difference between groups over time in specified brain region, ↓ indicate a decrease in the HR or CHR-T group compared to HV or CHR-NT respectively, ↑ indicate an increase and ↔ indicate no change over time in HR whereas an increase is seen in HV. HR, high risk; HV, healthy volunteer; CHR-T, clinical high risk transition; CHR-NT, no transition; NA, Not Applicable; Vol, white matter volume; MD, mean diffusivity; FA, fractional anisotropy; SLF, superior longitudinal fasciculus; FA

*, *lower in HR vs. HV at both timepoints but no group × time interaction*.

Most tract-based spatial statistics (TBSS) and tractography studies focused their analysis on regions of interest (ROI) rather than on the whole brain, finding a reduction or no change in FA in HR groups, whereas FA increased over time in HV; 2 studies examined the cerebellar tracts (61, 62), and 1 study measured tracts connecting the thalamus and hippocampus (60). For whole brain TBSS analyses, mean FA values reduced over time in GHR but increased for HV in the whole brain, the right cingulum, and the left posterior thalamic radiation, with a smaller increase in the right retrolenticular part of the internal capsule (63). Two whole brain TBSS studies did not find altered neurodevelopmental trajectories in HR, but instead found lower FA at baseline (59) or through both timepoints (65). Of these, Krakauer et al. (59) reported reduced FA in the right anterior thalamic radiation (ATR), left corticospinal tract (CST) and left superior longitudinal fasciculus (SLF), and Roalf et al. (65) reported lower FA in the CST and in the cingulum bundle of the hippocampus (CGH). Lower FA at baseline in the SLF may normalize over time, as another TBSS study found that FA in this tract increased over the course of 1 year in UHR subjects (59). A study in an older age cohort (mean age 76 years) found that polygenic risk score for schizophrenia correlated with increased mean diffusivity over time in the splenium, arcuate, ATR and cingulum, which may reflect reduced neuropil or increases in cerebrospinal fluid (55).

### Longitudinal Brain Trajectories in Subjects That Transition to Psychosis

For studies of gray matter comparing HR groups based on transition status (pre vs. post), 15 out of 19 studies report altered gray matter trajectories in those whose symptoms persist or transition to psychosis compared to those whose symptoms remit or those who do not transition to psychosis. Significant findings centered on the temporal (9 studies) and frontal cortex (6 studies), with fewer findings in the parietal lobe and anterior cingulate cortex (4 studies) (Table 3). Studies consistently reported greater reductions in gray matter volume, cortical thickness and surface area over time in those who remained symptomatic or transitioned to psychosis, with the exception of one study (29). In this study both resilient and non-resilient CHR individuals showed decreased cortical thickness over time in the superior temporal cortex and posterior cingulate gyrus, which started out lower in non-resilient subjects, however, the non-resilient group showed a slower rate of change than the resilient group. Furthermore, in the anterior cingulate cortex the study reported greater volume reductions over time in the non-resilient CHR group but an increase in gyrification over time. In general, studies comparing transition and non-transition groups alongside HV found greater cortical thinning in frontal and temporal lobes, as well as the caudate and insula, which were not apparent in the non-transition group when compared to HV (17, 20, 28, 32, 43, 44, 52).

For studies in CHR (rather than GHR), 11 out of 13 studies report altered trajectories in subjects who were most unwell. No studies in subjects with PE examined transitioned patients, although one study subtyped PE subjects based on negative symptom severity, finding widespread gray matter loss in those with more severe negative symptoms (36). In GHR 4 out of 6 studies found significant longitudinal changes based on clinical presentation (5 studies from the Edinburgh High-Risk cohort). Differing results in GHR subjects from the Edinburgh High-Risk cohort depend on the applied MRI analysis method. The most reliable results are described by Bois et al. (33), which was one of two studies in this cohort to apply correction for multiple comparisons. In this study, cortical thickness did not change over time according to clinical status, but differed in the whole GHR group compared to HV. As GHR subjects were split into three clinical groups; well, symptomatic and transitioned, this cohort may have been under-powered to detect differences between groups, and only a small proportion of participants transitioned in this cohort (*n* = 8).

For white matter, only 5 studies examined whether brain development differs in those who transition to psychosis and those who do not (Table 4), and all found greater reductions in white matter volume or FA in the former group, principally in the corpus callosum (3 studies) and in white matter regions near the superior fronto-occipital fasciculus (SFOF) (2 studies). One study used DTI methods and found reduced FA over time in transitioned subjects in a cluster of voxels in the anterior limb of the left internal capsule (left ALIC), the body of the corpus callosum (bCC), left superior corona radiata (SCR), and left SFOF (64), with an increase over time in non-transitioned subjects. Four studies measured white matter volume; 3 studies found reduced volume over time in the corpus callosum, left inferior frontal occipital fasciculus (IFOF), calcarine cortex, and whole brain of transitioned subjects (28, 45, 53). The other study of white matter found that corpus callosum volume reduced over time in all groups (healthy volunteers, resilient and non-resilient UHR) but that corpus callosum volume was highest in resilient UHR, intermediate in HV and lowest in non-resilient UHR (29). This contrasts with 2 studies reporting that the level of reduced white matter volume over time was intermediate in non-transitioned subjects between that seen in HV and transitioned subjects (28, 45), although no studies using DTI methods compared clinical subgroup trajectories to HV.

### Cross-Sectional MRI Differences at Baseline

Since the majority of studies find that “brain development” differs in HR groups compared to HV, with the most pronounced changes seen in subjects that transition to psychosis, this leaves open the question of whether differences are present cross-sectionally at the baseline timepoint. At baseline, 9 out of 14 studies report lower gray matter volume, cortical thickness, and surface area in HR subjects compared to HV in the frontal (5 studies), temporal (4 studies) and parietal lobe (4 studies) (Table 3). As more studies report longitudinal differences (20 out of 24), this suggests that longitudinal studies may have greater sensitivity to detect differences in gray matter. For white matter, a similar trend is seen, with 3 out of 9 studies finding lower fractional anisotropy at baseline between HR and HV, in the corpus callosum, CST, SLF, and ATR (Table 4), whereas 9 out of 13 studies detected longitudinal differences.

It is of interest whether baseline structural measures can predict clinical outcome in HR, as this could guide clinical interventions. At baseline, 9 out of 15 studies found lower gray matter volumes in those who went on to transition compared to those who did not, in the frontal (5 studies), parietal (4 studies), insula, and temporal lobe (3 studies). This is lower than the proportion of studies that detected longitudinal differences between clinical subgroups (15 out of 19 studies). For baseline measures of white matter, 3 out of 6 studies found higher white matter volume or FA in those who went on to transition compared to those who did not, with higher volume in the SLF and SFOF (53) and higher FA in the corpus callosum (57). Also in CHR higher FA in hippocampal-thalamic tracts was associated with more severe positive symptoms 12 months later (60). Taken together with the longitudinal findings in the left SFOF of transitioned subjects, it appears that those with higher FA values and white matter volume at baseline show the largest reductions over time. The proportion of studies finding white matter differences at baseline is lower than the proportion of studies that detected longitudinal differences between transition and non-transition groups (5 out of 5 studies).

## Discussion

The aim of this systematic review was to examine whether neurodevelopmental trajectories differ between (i) high risk subjects (HR) compared to healthy volunteers (HV), and (ii) those who transition to psychosis compared to those who do not. HR subjects, regardless of subtype (CHR, GHR, and PE), show an accelerated decline in gray matter primarily in the temporal cortex, and also the frontal, cingulate and parietal cortex. In those who remain symptomatic or transition to psychosis, there was a greater reduction in these same brain regions. Altered gray matter trajectories may normalize by early adulthood in HR cohorts whose sub-threshold symptoms resolve, whereas brain abnormalities progress in those whose symptoms do not remit. White matter volume and diffusion anisotropy, which usually increase until early adulthood, did not change or were reduced in HR subjects, principally in the cingulum and thalamic radiation amongst other regions including the cerebellum, retrolenticular part of internal capsule, and tracts connecting the hippocampus and thalamus. In subjects who transitioned to psychosis, there was reduced FA or volume principally in the superior and inferior fronto-occipital fasciculus and the corpus callosum, as well as the anterior limb of the left internal capsule, superior corona radiata and calcarine cortex.

In normal development, gray matter volume reduces from late childhood until the early twenties as a result of synaptic pruning and increased intra-cortical myelination (9, 66). Gray matter declines first in somatosensory areas, and last in higher-order association areas such as the dorsolateral prefrontal cortex, inferior parietal, and superior temporal gyrus (67, 68). This review suggests that latent vulnerability to schizophrenia is associated with premature gray matter loss in multiple higher-order brain regions. Diverging gray matter trajectories in the frontal and temporal cortex of HR populations are consistent with gray matter changes seen in schizophrenia (69, 70). In studies which include a patient group, reduced gray matter in HR appear to be intermediate between changes seen in schizophrenia and HV (23, 24), although one study reports a similar decline in cortical thickness in both patients and their healthy co-twins (27) and one study reports reduced hippocampal volume in schizophrenia patients but not GHR subjects (25).

**Figure 2:**
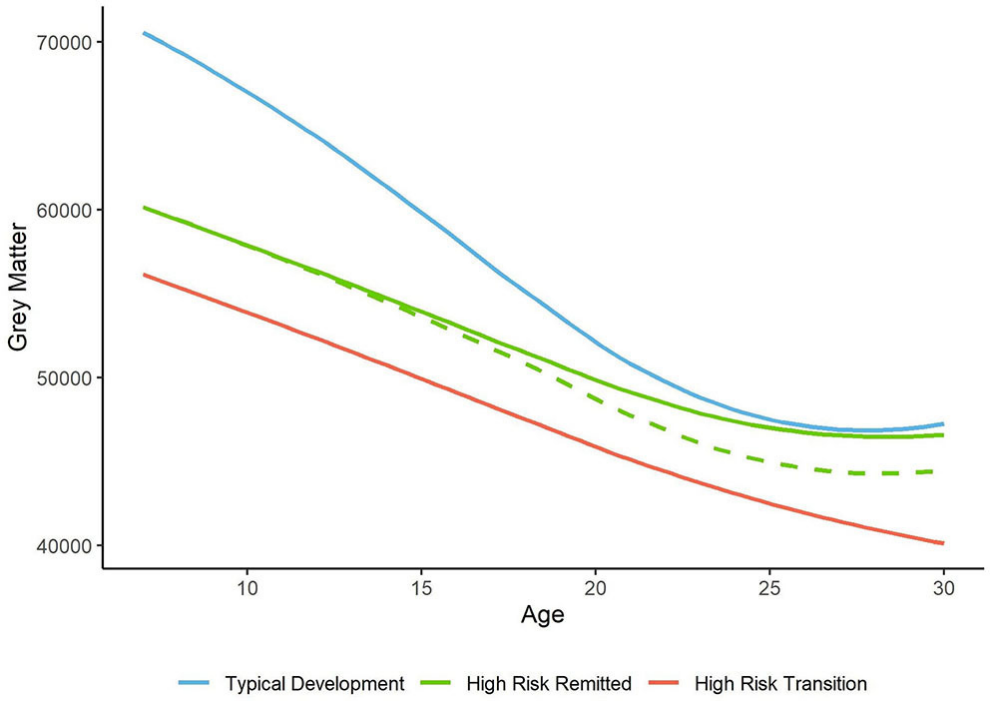
Hypothetical trajectories of whole brain gray matter. In typical development, gray matter volume and thickness reduce in late childhood, and plateau in mid adulthood [blue line, based on (9)]. In high risk subjects, gray matter volume and thickness is lower than healthy volunteers. In those whose symptoms improve this deficit may persist (dashed green line) or recover (solid green line). In those who transition to psychosis, lower gray matter is found at baseline and longitudinally compared to non-transition subjects (red line).

Brain development differs in those who go on to transition from those who do not. Although greater reductions in temporal, frontal, cingulate, and parietal lobe gray matter are observed in HR subjects as a whole, more pronounced changes in these same regions are observed in those who transition to schizophrenia, particularly in the temporal cortex. A number of hypothetical neurodevelopmental models may explain the diverging gray matter trajectories in HR subjects depending on clinical outcome (Figure 2). For HR subjects that do not transition, gray matter volume may initially be lower than HV, but during adolescence non-transition subjects have a slower trajectory of typical gray matter loss, allowing typically developing subjects to “catch up” to the same level of gray matter volume, as shown in the NIMH cohort and the Barcelona cohort (21, 52). This infers premature gray matter loss in HR or a neurodevelopmental deficit in gray matter volume, which no longer differs from HV later on in development. Alternatively, remitted HR subjects may retain this deficit in gray matter volume, but to a lesser extent than that seen in subjects who transition to psychosis. The former is most likely, as studies generally do not find an intermediate level of gray matter loss in remitted subjects compared to HV and transitioned subjects (17, 20, 28, 43, 44, 52). Cognition may be affected in HR subjects whose brain trajectories later “normalize,” as initial gray matter deficits alongside protracted loss are associated with poor intelligence (71), which is reported to be lower in GHR groups (72).

Gray and white matter differences between transition and non-transition subjects were more likely to be detected using longitudinal measures than baseline measures, and thus may offer predictive value to guide clinicians' care. Recent studies infer that cross-sectional brain structure measures offer additional predictive value to using clinical ratings alone (14, 73). Moreover, machine learning of “brain age,” the deviation between chronological and neuroanatomical age, has successfully identified CHR subjects from HV (74), further supporting the presence of accelerated brain aging in these subjects. To date, these multivariate models have not included cross-sectional or longitudinal DTI measures or longitudinal measures of gray matter. This review indicates that longitudinal studies better capture subtle changes in brain maturation, and so these measures may offer a higher degree of prediction accuracy.

**Figure 3:**
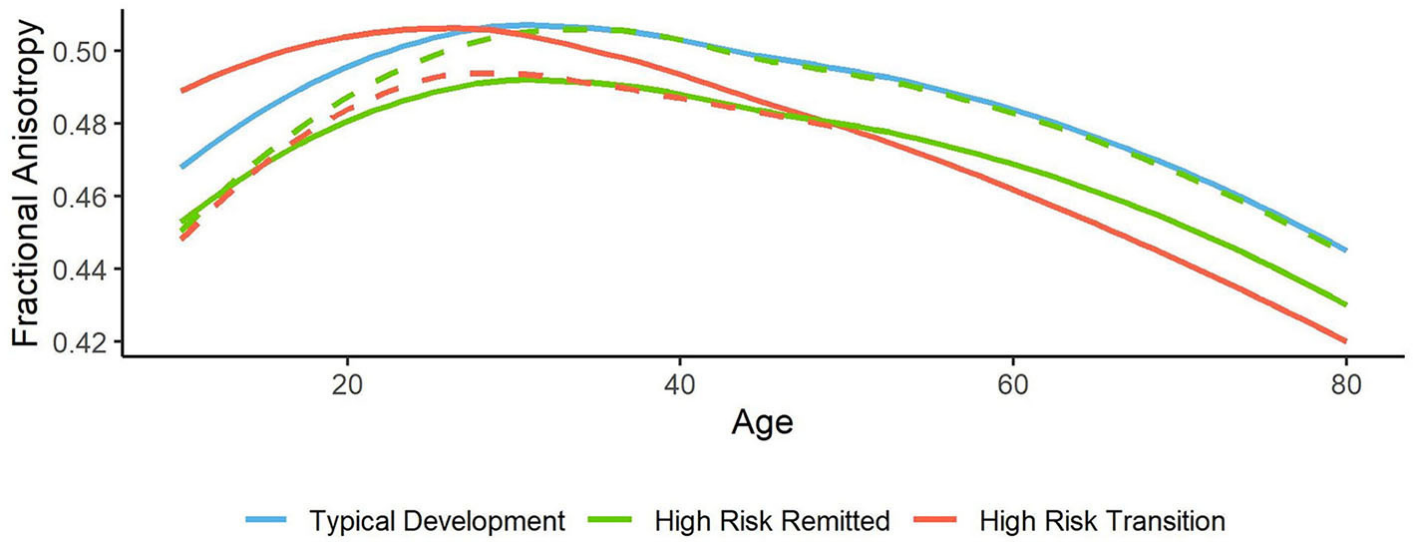
Hypothetical trajectories of whole brain white matter. For typical white matter development, fractional anisotropy values peak in mid adulthood, and reduce around age 40, with a steep decline after age 60 [blue line, based on (77)]. Lower FA values are observed in high risk subjects; which may be stable over time (green line), or reflect a neurodevelopmental delay which recovers over time (dashed green line). In patients who transition, pre-mature development and aging may occur (red line), as some studies find higher FA levels in CHR-T subjects at baseline and a greater reduction over time. Alternatively those who go on to transition may possess initial deficits which later progress further than non-transition subjects (red dashed line).

White matter volume and diffusion anisotropy (measured by FA) typically increase until early adulthood, however in HR these measures did not change, reduced over time or showed smaller increases compared to HV. ROI analyses in HR reported reduced FA over time in the cerebellum and tracts connecting the thalamus and hippocampus (60–62), whereas whole brain analyses most commonly found reduced FA in the thalamic radiation and cingulum in HR subjects compared to HV (55, 63, 65). Some studies did not find an interaction between group and time, and instead report lower FA at both timepoints in the SLF, ATR, corticospinal tracts, and the cingulum (59, 65). The absence of an interaction may occur when FA values stabilize in HV in mid-adulthood. Reduced FA is consistent with findings in schizophrenia, which are widespread throughout the brain, with the strongest effect in the anterior corona radiata and corpus callosum (75, 76). When psychotic disorder patients were examined alongside HR groups, baseline FA was lowest in patients, intermediate in CHR and highest in controls (64). Psychotic disorder patients had lower whole-brain mean FA at both timepoints, whereas FA reduced over time in GHR and slightly increased in HV (63). This suggests accelerated aging begins in prodromal individuals, which creates a later deficit when psychosis emerges. Depending on age, lower FA may represent delayed neurodevelopment (before mid-adulthood) or premature aging (after mid-adulthood) (Figure 3). Another possible interpretation would be an overdevelopment of secondary white matter pathways in HR, causing reduced FA in regions with increased crossing fibers.

It is unclear whether altered white matter development “recovers” in HR, as although one study found that slower white matter growth normalized by age 14 (54), the majority of studies detected reduced FA and volume after this age. Instead the level of white matter volume reduction may be intermediate in non-transitioned subjects between that of transitioned subjects and HV (28, 45), meaning that non-transitioned subjects show a neurodevelopmental deficit which may not recover. Alternatively, white matter volume or FA may increase in non-transitioned subjects as a compensatory process; one study found that FA increased over time in these subjects but reduced in those who transitioned (64), and one study reported that corpus callosum volume reduced over time in all groups, but was consistently larger in resilient UHR, intermediate in HV and lowest in non-resilient UHR groups (29). Therefore, further work is needed to establish whether low FA in HR represents a stable deficit, accelerated aging or a neurodevelopmental delay which recovers.

In those who transition, reduced volume or FA over time was principally reported in the IFOF, the SFOF and the corpus callosum, as well as the anterior limb of the internal capsule and superior corona radiata, which are white matter regions showing the strongest alterations in schizophrenia (76). Cross-sectional studies report higher white matter volume and FA at baseline in those who went on to transition compared to those who did not, in a number of brain regions (57, 60) including the SFOF (53). If white matter integrity then reduces over time, this could suggest premature development and aging of white matter in transitioned subjects. Although longitudinal studies assessed here generally found higher FA at baseline, this is in contrast with the wider cross-sectional literature (11). Further studies in those who go on to transition are needed to conclude whether FA is initially higher (premature development) or lower (neurodevelopmental deficit), which then progressively reduces beyond that seen in non-transition subjects (neurodegenerative) (Figure 3).

There are significantly fewer longitudinal studies investigating white matter compared to gray matter in HR, in particular those examining white matter in transition and non-transition subjects, which were limited to small sample sizes (ranging from *n* = 5 to *n* = 18). Eight studies measured white matter volume, which (a) has less anatomical specificity than tractography and (b) are inherently coarser measures than the microstructural metrics offered by DTI. Ten studies used DTI, half of which focused on ROI or specific tract analyses rather than whole-brain approaches, which may not provide a complete picture of white matter differences between groups. The specificity of the observed white-matter changes is limited, even with DTI, as they are often reported in regions of overlapping tracts and complex white matter microstructure. For example, the reported alterations in transitioned subjects point toward areas of crossing or overlapping fibers in the frontal lobe, which these studies have referred to as the SFOF. The existence of the SFOF in humans has been disputed (78–80), and the results from these studies may instead refer to corticostriatal and thalamic peduncle fibers interconnecting the cortex with the striatum and the thalamus. More advanced diffusion-weighted MRI techniques based on multi-shell MRI acquisition sequences can derive more detailed measures of the cellular environment, and will help to disentangle which specific tracts underlie the white matter changes (81).

The studies included in the systematic review have a number of limitations. Firstly, the majority of studies include participants who are taking antipsychotic medication. Treatment with antipsychotics is associated with reductions in global gray matter volume and enlarged lateral ventricles (82). This can bias results, particularly if the majority of the transition group are medicated whereas the non-transition group are not (41). However, in the largest study to date, reductions in cortical thickness in those who transitioned remained significant when analyses were restricted to unmedicated subjects (17), whilst another study found trend-level associations after excluding medicated subjects (52). Future studies could make use of sampling psychotic experiences in the general population, which were examined by 5 studies in this review. These samples are not help seeking, and thus rarely receive antipsychotic medication.

The majority of studies were of low to medium quality, and a third of studies examining gray matter did not correct for multiple comparisons. As changes in HR are more subtle than those seen in schizophrenia, it is recommended that future studies use a priori small volume correction (SVC) in regions of interest, such as the medial temporal lobe. For example, one study detected gray matter loss in those with psychotic experiences compared to those without, but only when SVC was applied in the temporal lobe (35). Brain differences in HR are not global, or localized to discrete regions, but instead appear to affect certain networks such as the fronto-parietal, and limbic-temporal networks. Although SVC will increase the power to detect subtle changes, these must be complemented with whole brain and network based approaches. Lastly, an important issue in the reporting of MRI studies is publication bias, which cannot be formally assessed by a systematic review. A number of studies examined brain changes over time in HR and HV groups separately, whereas future studies should report interaction analyses between group and time.

In conclusion, HR subjects show accelerated gray matter loss in the temporal, frontal, cingulate and parietal cortex. This is alongside reduced white matter volume and reduced FA over time in the thalamic radiation and cingulum, which may reflect a neurodevelopmental deficit in white matter maturation or premature aging. Over time gray matter trajectories may converge between HR and HV groups, but not in those who remain symptomatic or transition to disease. Transitioned subjects show progressive gray matter reductions in the temporal and frontal lobes, and reduced white matter in the SFOF, IFOF, corpus callosum, and corona radiata, on a continuum with that seen in schizophrenia. Longitudinal gray and white matter measures were more likely to detect differences between transition and non-transition subjects than baseline measures, and thus may offer predictive value to guide clinicians' care. The availability of advanced neuroimaging technologies will allow future studies to better track white matter changes in the prodrome, which may offer promising markers of clinical outcome.

## Data Availability Statement

The original contributions generated in the study are included in the article/Supplementary Material, further inquiries can be directed to the corresponding author.

## Author Contributions

KM, PL, and AI: extracted data. KM, PL, and AD: wrote the manuscript. All authors: contributed to the article and approved the submitted version.

## Conflict of Interest

The authors declare that the research was conducted in the absence of any commercial or financial relationships that could be construed as a potential conflict of interest.
