# Supplementary material for: Longitudinal Structural MRI Findings in Individuals at Genetic and Clinical High Risk for Psychosis: A Systematic Review
Source: Front Psychiatry. 2021 Feb 2;12:620401. doi: 10.3389/fpsyt.2021.620401 (PMC7884337; doi:10.3389/fpsyt.2021.620401)
Supplement: Supplementary file 1 [file Table_1.docx]

# Supplemental Material

eTable 1……………………………………………………………………………………………………………………………….……………2

eTable 2……………………………………………………………………………………………………………………………….………….10

eResults...………………………………………………………………………………………………………………………………………..17

eReferences.……………………………………………………………………………………………………………………………………24

**Table 1:** **Results summary of studies examining grey matter.** GHR: Genetic high risk, CHR: Clinical high risk, T: transition, NT: no transition, S: symptomatic, NS: no symptoms, FEP: First episode psychosis, MII: mild intellectual impairment, PE: Psychotic experiences, BL: Baseline FU: Follow-up, SIS: Structured Interview for Schizotypy, TBM: tensor based morphometry, SVC: small volume correction, FDR: false-discovery rate correction, FWE: familywise errors correction, L: left, R: right, NS: Not Significant, ACC: anterior cingulate cortex, STG: superior temporal gyrus, mPFC: medial prefrontal gyrus, FA: Fractional anisotropy

| Dataset | Authors | Risk Group | HV | Outcome measure | Correction | Analysis | Contrast | Longitudinal Results | Cross Sectional results |
| --- | --- | --- | --- | --- | --- | --- | --- | --- | --- |
| Toho University | Katagiri, 2019 | 42 CHR:  5 T  37 NT:  (Further divided  23 med  14 unmed) | 16  BL only | ROI: 3 striatal subregion volumes + intracranial volume.  FreeSurfer | ✘  4 regions examined | 1) RM-ANOVA medicated + unmediated CHR-NT.  2) multiple regression ΔPOS and ΔNEG symptoms + ΔROI | 1) NT med vs NT unmed  2) CHR NT | Reduction in putamen in NT med and NT unmed (effect of time)  2) Positive symptom recovery w/ accumbens increase | HV vs CHR-T vs NT-med vs NT-unmed:  NS striatum between groups. |
| ADAPT Program, Northwestern University | Damme, 2019 | 81 CHR | 38 | Gyrification (lGI), shape of the curve (MCI), sulcal depth (SD).  FreeSurfer 6.0 | FDR-corrected P=0.05 | 1) MCI + SD: RM-ANOVA  2) interclass correlation of significant clusters from (1) to assess gyrification stability over time | CHR vs HV | 2) No change in cortical surface morphometry over time in all or in CHR | 1) numerous differences in surface measures (CSM) in cingulate, frontal, temporal, occipital and parietal cortex |
| NAPLS | Cannon, 2015 | 274 CHR:  35 T,  239 NT | 135 | Cortical thickness  FreeSurfer 5.2 | FDR-corrected P=0.01 | Annualized rates of percent change (ARCH).  1) Whole brain  2) ROI: PFC & ventricles | CHR-T vs CHR-NT vs HV | 1) Greater thinning in CHR-T:  R superior frontal, middle frontal, and medial orbitofrontal. Greater expansion in 3^rd^ ventricle.  No results in left hemisphere  2) Unmedicated CHR-T: greater reduction in R PFC thickness and greater expansion of ventricle. Did not differ from medicated CHR-T | NS at baseline between converters, non-converters, and HV |
|  | Chung, 2015 | 274 CHR:  35 T,  239 NT | 135 | Cortical thickness  Monte-Carlo simulation corrected. FreeSurfer 5.2. | Monte Carlo simulations | Annualized rates of  change + P1 symptoms  1) Whole brain  2) ROI: 3rd ventricle | Regression in groups separately:  HV  CHR-NT  CHR-T | 1) CHR-T: higher P1 symptoms at BL associated with steeper grey matter decline in L+R superior + rostral middle frontal gyrus  2) CHR-T: higher BL P1 symptoms associated with ventricle enlargement  3) In HV or CHR-NT no association with symptoms | Cannon et al., 2015a  BL GM not associated with P1 symptom ratings |
|  | Chung, 2017  Addition of 3^rd^ timepoint | 267 CHR:  37 T,  230 NT | 132 | Cortical thickness. FreeSurfer. | FDR-corrected P=0.01 | Δ gm + Δ ventricle  1) GLM Whole brain.  2 + 3) ROI superior frontal cortex (SFC) | 1) CHR + HC separately  2) CHR-NT vs CHR-T  3) HV vs CHR | 1) Whole brain, CHR only: ventricle expansion associated with cortical grey matter thinning. Less widespread in HC  1) In ROI SFC: Interaction with above analysis. Relationship between ventricle + gm in CHR but not controls  2) converters showed relationship, non-converters + HV did not.  3) 1yr-2yr timepoints showed same relationship as BL to 1yr timepoints in CHR, but not HV for i) whole brain and ii) ROI SFC | Cannon et al., 2015a |
| Singapore Longitudinal Youth At-Risk Study | Ho, 2017 | 93 CHR:  41 Non Remit (NR),  52 Remit (R) | 54 | ROI: hippocampus 7 subfields including CA1.  FreeSurfer 5.3. | Holm–Bonferroni for 7 regions | 1) LMM baseline for each region  2) LMM group x time  3) Regression ΔCAARMS and change in sig regions from (2), CHR-NR only | Non Remit (CHR-NR) vs CHR-Remit (CHR-R) vs HC | 2) CHR-NR vs R (+ HV): decline right global hippocampus, bilateral CA1 & right CA3  3) CHR-NR: decline in CA1 associates with increasing CAARMS severity. CA3 NS. | 1) NS |
| NIMH COS Study | Gogtay, 2007 | 52 GHR | 52 | Cortical thickness (in-house method) | FDR correction P=0.05 | 1) Mixed-model regression with age  2) Regression with global functioning in GHR | GHR vs HV | 1) Reduced GM CT left prefrontal and bilateral temporal cortices, from age 8, normalised by age 20. HC steeper slope of GM loss. Small region parietal GM loss normalised by age 17.  2) thickness increased with functioning | NS average of all scans |
|  | Mattai, 2011  Independent sample to Gogtay 2007 | 43 GHR | 86 | Cortical thickness (in-house method) | FDR correction P=0.05 | Linear mixed effects regression: volume with group, age, group x age. | GHR vs HV | In GHR, reduced GM in bilateral prefrontal and L temporal cortices. Smaller deficits in parietal & right inferior temporal. Normalised by age 17. Steeper trajectory of loss in HV | NS average of all scans |
|  | Zalesky, 2015  Same sample as Gogtay 2007 plus 39 patients and 34 siblings | 86 GHR  109 Child-onset SZ | 102 | Cortical thickness of 33 regions per hemisphere, clustered to form 5 lobes: frontal, parietal, temporal, occipital, and cingulate | FWE-correction P=0.05 | Corticocortical connectivity determined by Pearson correlation in cortical thickness between pairs of regions or lobes. | GHR vs HV | In GHR, reduced correlation between occipito-temporal areas (ie reduced connectivity), which normalized by age 17 (delayed maturation). Childhood-onset SZ patients normalise only by age 24. | - |
|  | Greenstein, 2011 | 80 GHR  94 Child-onset SZ | 110 | ROI: Cerebellum. Parcellation BRAINS2 | ✘ | Polynomial mixed model regression | GHR vs COS vs HV | In GHR: reduction in total cerebellum, left inferior posterior, left superior posterior lobe, and total right hemisphere volumes vs HV over time. Does not normalise, but greater superior vermis volume converged with HV trajectory over time. GHR and COS trajectories diverged only in left anterior lobe. | GHR smaller R cerebellar & left superior posterior cerebellum than HV |
|  | Mattai, 2011 | 78 GHR  89 Child-onset SZ | 79 | ROI: Total, 1 volumes.  FreeSurfer | ✘ | Linear mixed-effects regression | GHR vs COS vs HV | No change in hippocampal volume over time between groups | NS |
| Sibling pair cohort, Utrecht | Brans, 2008 | 11 GHR  11 SZ patients | 33 | Volume: whole brain, cerebral grey and white matter, cerebellum, lateral & third ventricles. | ✘ | Mixed models | SZ vs siblings (GHR) vs HV | GHR vs HV: No significant differences in volume over time  (Whole brain volume & cerebral GM decreased excessively in SZ patients) | - |
| Twin pair cohort, Utrecht | Hedman, 2016 | 19 GHR (twin pairs)  9 MZ:  10 DZ discordant | 54:  14 MZ 13 DZ | Whole brain + multiple ROI. Cortical thickness and surface area. CLASP | 39 regions, 43 IV, bonferroni = P<1.19 x 10^−3^ | Repeated-measures ANOVA of change per year data | SZ vs Co-twin vs Control twins | Patients + co-twins showed reduction in global cortical thickness vs HV twins  ROI: co-twins vs HV twins reduced thickness in L superior temporal cortex.  Surface area NS. | - |
| Dutch Prediction of Psychosis  Study, Utrecht | Ziermans, 2012 | 43 CHR:  8 T  35 NT | 30 | 1) Semi automated volumes for total brain, lateral ventricles, cerebellum, GM  and WM.  2) Cortical thickness CLASP  3) VBM volume. | FDR correction P=0.05 for (2) + (3)  None for (1) | 1) Linear regression. Change in volume per year  2 + 3) vertex by vertex GLM | CHR vs HV  CHR-T vs CHR-NT vs HV | CHR vs HV:  CHR greater reduction cortical thickness L middle temporal vs HV  CHR-T vs CHR-NT vs HV:  1) CHR-T: greater total brain volume loss than CHR-NT + HV  2) CHR-T vs HV: cortical thinning L ACC, precuneus, temporo-parietooccipital area  2) CHR-NT vs HV: NS cortical thickness  3) NS all groups VBM | NS volume + cortical thickness + GM or WM density. Trend reduced intracranial volume in CHR-NT. |
|  | De Wit, 2016 | 35 UHR:  17 Resilient  18 Non- Resilient UHR | 24 | Average  volume, cortical thickness, surface area, 34 cortical structures in each hemisphere + cerebellum. FreeSurfer 5.1.0 | ✘ | Linear mixed model; relationship between age, group and brain measures. | UHR vs HV  Resilient vs non-resilient UHR | UHR vs HV: less steep decreases in surface area over time in UHR in frontal and parietal areas. Hippocampus and thalamus volume smaller in UHR (effect of group) with steeper decreases over time (interaction). Volume of third and inferior lateral ventricle smaller at baseline with steeper increases over time in UHR.  Resilient vs non-resilient UHR, effect of group: Resilient individuals had larger volumes of frontal, temporal and parietal cortex, corpus callosum and nucleus accumbens. Also greater cortical thickness in frontal, parietal and temporal lobes and larger cortical surface area in the left hemisphere in resilient UHR.  HV trajectories were between that of resilient and non-resilient UHR  Interaction:  Resilient UHR: smaller volume decrease over time in anterior cingulate gyrus and smaller increase in lateral ventricle volume. Increased surface area over time in precentral gyrus and frontal pole compared to decreases in non-resilient UHR.  Cortical thickness lower in non-resilient UHR at baseline in superior temporal cortex  and posterior cingulate gyrus, over time a greater reduction in these regions in resilient UHR, and decreases in gyrification in anterior cingulate gyrus over time compared to increases in non-resilient UHR (although ACC volume is reduced in non-resilient UHR). | - |
| Edinburgh High-Risk Study | Lawrie, 2002 | 66 GHR:  19 Symp  47 NS | 20 | ROI: amygdala-hippocampus & temporal lobe volume.Semi-automated tracing | ✘ 9 regions examined, voxel wise analyses not conducted | Repeated-measures ANOVA | GHR vs HV  Symptomatic vs non-symptomatic | NS GHR vs HV.  Symptomatic GHR reduced R temporal lobe volume vs non-symptomatic GHR. | (83) GHR vs HV reduced volume amygdalo-hippocampus & thalamus |
|  | Job, 2005 | 65 GHR:  18 Symp  47 NS | 19 | Whole brain + ROI SVC: amygdala-hippocampus volume.  VBM SPM | Correction used, not specified. | 1) Repeated-measures ANOVA GHR vs HV.  2) S vs NS.  3) t-test for each group over time, masking results from other group 4) repeat with n=8 transition | GHR vs HV  Symptomatic vs non-symptomatic  GHR only  Transition only | 1) NS: GHR vs HV  2) NS: GHR-S vs GHR NS  3) In GHR; GM reduction in temporal lobes, R cingulate gyrus, R parietal lobe & cerebellum (L amygdala unmasked)  4) In transition: reduced GM in L temporal lobe & R cerebellum | - |
|  | Mcintosh, 2011 | 146 GHR:  17 T,  72 NT,  57 Symp | 36 | Whole brain volume + ROI: prefrontal & temporal lobe. Semi-automated tracing | ✘ 5 regions examined. | Linear mixed model | GHR vs HV  GHR-T vs GHR-NT vs GHR-S | 1) GHR vs HV Reduced whole brain volume and L&R prefrontal and L temporal lobe  2) Reduced prefrontal in GHR-T vs other GHR groups. NS temporal. | GHR vs HV: Smaller L+R prefrontal lobes  Clinical subgroups NS |
|  | Bois, 2015 | 142 GHR  (64 FU):  17 T (8 FU)  68 NT (30 FU)  57 Symp (26 FU) | 36  (18 FU) | Cortical thickness and surface area. Global & ROI: Frontal, temporal, cingulate, insular, parietal, & occipital lobes.  FreeSurfer 5.3. | FDR-corrected | Linear mixed models | GHR vs HV  GHR-T vs GHR-NT vs GHR-S | GHR preserved global surface area whereas reduced in HV. NS when SA was examined regionally in each lobe.  GHR reduced cortical thickness over time, globally and frontal & cingulate, whereas HV showed increase, globally and in occipital.  No difference between clinical subgroups: GHR-T vs GHR-NT vs GHR-S | NS GHR vs HV.  GHR-T had a significantly larger surface area than GHT-NT for global but not lobar measures. |
|  | Bois, 2016 | 142 GHR  (64 FU):  17 T (8 FU)  68 NT (30 FU)  57 Symp (26 FU) | 36  (18 FU) | ROI: hippocampus & amygdala GM volume independently (prev hippocampal–amygdala complex). FreeSurfer 5.3. | NA: 2 ROIs examined | Linear mixed models | GHR vs HV  GHR-T vs GHR-NT vs GHR-S | 1) Hippocampal volume increased in HV, no change in in GHR. NS amygdala.  2) NS group x time when GHR subgroups examined | - |
| Edinburgh Study of Comorbidity | Moorhead, 2009 | 53 MII + PE | 45 | GM + WM volume TBM  Whole brain + SVC: L temporal lobe & L amygdala-hippocampal complex. | FWE-correction P=0.05 | 1) VBM GLM  2) Spearman's rho Peak voxels for change in  grey matter tissue and change in symptoms | PE+ vs PE- | Whole brain: NS  SVC left amygdala-hippocampal complex:  PE+ greater GM loss in L ventral + medial amygdala & L parahippocampal gyrus vs PE-  SVC left temporal lobe: PE+ greater GM loss parahippocampal gyrus  Sig association between worsening symptoms and grey matter loss | - |
|  | McKechanie, 2016 | 43 MII + PE:  9 negative, 34 not | ✘ | TBM  SPM 5 | Cluster significance corrected for multiple comparisons  P=0.05 | SPM GLM | Negative symptoms vs no negative symptoms | Those with negative symptoms: Reduced GM in occipital, inferior parietal, posterior cingulate and the medial temporal lobes & cerebellum. Latter 2 survived when med excluded | NS between those with and without negative symptoms |
| FEPSY project  Basel, Switzerland | Walter, 2015 | 23 CHR  10 T  13 NT  Partial overlap with Walter 2012 | ✘ | ROI: Pituitary gland. Manual tracing | ✘ 1 region | Linear mixed models | CHR-T vs CHR-NT | NS: Pituitary volume change over time | (84) Larger pituitary in CHR-T vs NT |
|  | Walter, 2012 | 18 CHR  8 T  10 NT | ✘ | ROI: Hippocampus. Manual tracing | ✘ 1 region | Linear mixed models | CHR-T vs CHR-NT | NS group x time. Hippocampus volume decreased over time in both groups. | - |
|  | Borgwardt, 2008 | 20 CHR:  10 T  10 NT | ✘ | Whole brain volume VBM SPM5 | Cluster-forming threshold  p=0.001 uncorr. Cluster level at P=0.05 | ANCOVA: Follow-up minus baseline difference images for each group seperately | CHR-T  CHR-NT | CHR-T: volume reductions in the orbitofrontal, superior frontal, inferior temporal, medial and superior parietal cortex, and cerebellum  CHR-NT: No change over time | (85,86) CHR-T vs NT less GM in R insula, inferior frontal & superior frontal gyrus |
| OASIS, London | Fusar-Poli, 2011 | 22 CHR:  5 T  17 NT | 14 | Whole brain volume VBM SPM5 | FWE-correction P=0.05 | ANOVA on each group separately | HV  CHR  CHR-T  CHR-NT | 1) No change in HV.  In CHR reduced GM L+R superior frontal gyrus & R caudate.  Increased GM R inferior frontal gyrus, R anterior cingulate & left cerebellum.  2) CHR-NT reduced GM L+R superior frontal gyrus, R caudate & L putamen. Increase L+R inferior frontal gyrus & R anterior cingulate.  CHR-T reduced GM R middle temporal gyrus. | 1) CHR vs HV: smaller GM volume R superior frontal + superior temporal gyrus, L medial + inferior frontal gyrus & R orbital gyrus  2) T vs NT: smaller GM volume R inferior parietal lobule & L para hippocampal gyrus |
| PACE, Melbourne | Pantelis, 2003 | 21 CHR:  10 T  11 NT | ✘ | GM volume VBM | ✘ | 1) Within-group  repeated-measures ANCOVA  2) Group x time | CHR-T vs CHR-NT | CHR-T: reduced GM L parahippocampal, fusiform, orbitofrontal, cerebellar cortices & cingulate gyri. Increase R cuneus  CHR-NT: Reduced GM in similar region of L cerebellum  2) NS group x time | CHR-T vs NT: less GM R medial + lateral temporal, inferior frontal cortex (inclu BG and insula) & bilateral cingulate cortex |
|  | Sun, 2009 | 35 CHR:  12 T  23 NT  (20 included in Pantelis) | ✘ | Cortical Pattern Matching. Cortical surface motion analysis | 100,000 randomized permutations | Between groups t-tests of annualised contraction rate to create P-map | CHR-NT vs CHR-T | CHR-T vs NT: Reduced brain surface area R prefrontal region | - |
|  | Takahashi, 2009 | 35 CHR:  12 T  23 NT  23 FEP | 22 | ROI manual trace: superior temporal gyrus volume; (planum polare, Heschl gyrus, planum temporale, rostral & caudal regions) | ✘ 5 regions | 1) Repeated-measures ANCOVA for each region | HV vs CHR-NT vs CHR-T vs FEP  Did not assess CHR vs HC | FEP and CHR-T: GM reduction in planum polare, planum temporale, and caudal STG vs CHR-NT or HV | At FU, male CHR-T smaller planum temporale vs HV  At both timepoints, FEP smaller caudal STG than all other groups |
|  | Takahashi, 2009 | 31 CHR:  11 T  20 NT | 20 | ROI manual trace: insular GM volume. Whole, short &  long insular cortices | ✘ 2 regions | Repeated-measures ANCOVA of percent volume change | HV vs CHR-NT vs CHR-T  Did not assess CHR vs HC | CHR-T reduced GM in insula vs CHR-NT or HV.  (No GM change in CHR-NT and HV) | CHR-T smaller L+R insular cortex vs CHR-NT, + HV (R only) |
| Ludwig-Maximilians-University, Munich | Koutsouleris, 2010 | 25 CHR:  12 T  13 NT | 28 | GM + WM volume  Deformation-based  morphometry pipeline (DBM). SPM5 | 5000 permutations | VBM5 + partial-least-squares (multivariate, data-driven approach) | 1) HV vs CHR  2) CHR-T vs CHR-NT | 1) Morphometric abnormalities in R prefrontal, L+R perisylvian (including insular cortex + superior temporal gyri), cingulate, parietal, occipital, caudate nucleus, the cerebellum, vermis & periventricular structures.  2) Abnormalities more pronounced in CHR-T vs CHR-NT; middle and posterior cingulate cortex, medial  parietal cortex & adjacent ventricular space | (87) CHR-T smaller prefrontal grey matter volume vs CHR-NT or HV |
| Western Psychiatric Institute and Clinic, Pittsburgh | Bhojraj, 2011 | 56 GHR | 36 | Freesurfer ROI:  auditory association areas (AAA) within STG.  Cortical thickness, surface area & lateralization. | ✘  1 region | Repeated measures ANCOVA | GHR vs HV | Left AAA surface area and surface area laterality-index reduced over time in GHR vs HV. (Left SA reduced over time, R increased over time in GHR) Most prominent in males.  Reduced cortical thickness in male GHR vs HV  Increased cortical thickness in female GHR vs HV | - |
|  | Bhojraj, 2011 | 23 GHR | 27 | Freesurfer ROI: inferior parietal lobule, rostral ACC, temporal pole, frontal pole, inferior frontal gyrus, mPFC, orbitofrontal cortices. | ✘  Result did not survive correction for multiple comparisons | 1) Repeated measures ANCOVA  2) Spearman's correlations Δsymptoms and Δvolume of significant ROIs from (1) | GHR vs HV | 1) GHR volume declined, HV increased or remained stable for bilateral lateral orbitofrontal, L rostral anterior cingulate, L medial prefrontal, R inferior frontal gyrus & L temporal pole  2) Greater longitudinal reductions predicted worsening of symptoms over time | Smaller bilateral amygdalae,  pars triangulares, L lateral orbitofrontal, R frontal pole, angular & supramarginal gyri in GHR vs HV  Smaller BL volumes in L amygdalar, L inferior frontal gyrus and R angular gyrus volumes predicted greater  prodromal symptoms at FU |
|  | Prasad, 2010 | 31 GHR:  16 FU | 33:  15 FU | Cortical surface area, curvature and thickness using BRAINS2  Total volume of each lobe (grey, white and CSF volumes) | ✘ 5 regions | Cross-sec: mixed models: group, group ×  type (gyral or sulcal), group by hemisphere and group × lobe × hemisphere interactions.  Longitudinal: linear mixed-effects models. | GHR vs HV | GHR: reduction in total + frontal + occipital surface area (SA), whereas SA increased over time in HV.  SA NS in parietal + temporal.  GHR cortical thickness increase in temporal + parietal lobes. In HV CT decreased over time in these regions.  CT NS in frontal + occipital.  GHR: increase curvature in temporal lobe vs HV.  Greater grey matter loss in GHR vs HV in all lobes. | Parietal gyral surface area  & cortical thickness were reduced in GHR vs HV.  Parietal sulcal curvature was increased in GHR vs HV.  Frontal gyral surface area was reduced in GHR vs HV. |
| IMAGEN | Yu, 2020 | 706 scored on PE questionnaire | ✘ | ROI: hippocampus. Deformation-based morphometry VBM8 + SPM12b | FWE correction P=0.05 | Multiple linear regression  models: rate of GM volume change + CAPE total score of PE | - | Higher CAPE total score (PE) is related to a smaller expansion in the right uncus. |  |
| Dublin & Kildare | Calvo, 2020 | 25 PE | 25 | Hippocampal subfield volumes, combined to yield total hippocampal volume. FreeSurfer v6.0 | ✘ 1 region | Mixed-model repeated measures | PE vs HV | NS group x time. Significant effect for group: PE lower L+R whole hippocampal volumes. | PE lower L+R whole hippocampal volume at both timepoints. |
| Barcelona | Sugranyes, 2017 | 58 GHR | 34 | Cortical thickness (CT), surface area (SA) (34 cortical regions) plus mean lobar brain measures for grey matter volume. FreeSurfer | FDR correction P=0.05 | Linear mixed-effects models | GHR vs HV  Split into pre-pubertal and pubertal groups | Smaller decrease in global SA in GHR than HV  Smaller decrease in mean CT in GHR than bipolar offspring | CT: NS  GHR smaller global, parietal, and occipital lobe SA at baseline compared with HV. |
|  | Sugranyes, 2020 | 79 GHR:  20 Symp  59 NS | 49 |  |  |  | GHR-S vs GHR-NS vs HV | CT: Sig group x time; greater mean CT loss over time in GHR-S vs both GHR-NS and HV. Greater loss in occipital lobe in GHR-S vs GHR-NS, and trend for GHR-S vs HV in frontal lobe  SA: [GHR-S have smaller total SA at all timepoints] Smaller decrease in GHR-NS vs HV in total SA and in the parietal lobe.  Vol: GHR-NS had smaller decrease in total grey matter volume than HV | CT: NS  SA: smaller total SA in GHR vs HV. Smaller total SA in GHR-S vs HV and GHR-NS. At a lobar level, there was a trend for smaller SA in GHR-S vs HV in frontal, temporal &  parietal lobes, and in GHR-S vs GHR-NS in temporal  lobes.  Vol: GHR-S smaller total + parietal lobe grey matter volume than HV and GHR-NS. |

**Table 2: Results summary of studies examining white matter.** SZ: schizophrenia: GHR: genetic high risk, CHR: Clinical high risk, T: transition, NT: no transition, FEP: First episode psychosis, PE: Psychotic Experiences, szPGRS: Schizophrenia polygenetic risk, HV: Healthy Volunteers, BL: Baseline, FU: Follow-up, WM: white matter, GM: grey matter, CSF: cerebrospinal fluid, GML: general linear model, TBM: tensor-based morphometry, TBSS: tract-based spatial statistics, TFCE: threshold-free cluster enhancement, ICC: Intra-class correlation coefficient, FDR: false-discovery rate correction for multiple statistical comparisons, FWE: familywise errors correction for multiple statistical comparisons, SVC: Small volume correction, SEM: structural equation model, L: left, R: right, FA: Fractional anisotropy, NS: Not Significant, CGH: cingulum bundle of the hippocampus, LPC: lateral prefrontal cortex, MCC: medium cingulate cortex, PCC: posterior cingulate cortex, CST: corticospinal tract, AF: arcuate fasciculus, ATR: anterior thalamic radiation, PTR: posterior thalamic radiation, IFOF: inferior fronto-occipital fasciculus SFOF*: superior fronto-occipital fasciculus, ILF: inferior longitudinal fasciculus, SLF: superior longitudinal fasciculus, SCP: superior cerebellar peduncle, MCP: middle cerebellar peduncle, LCP: left cerebral peduncle, UF: Uncinate Fasciculus

*The existence of the SFOF in humans has been disputed (58–60).

| Dataset | Study | Risk Groups | HV | Main MRI outcome derivative | Cross-sectional Analysis | Longitudinal Analysis | Analysis details | Cross Sectional Results | Longitudinal Results |
| --- | --- | --- | --- | --- | --- | --- | --- | --- | --- |
| Sibling pair cohort | Brans 2008 | • 11 SZ  • 11 GHR siblings | 33 | Cerebral WM volume | ✘ | Group effect on yearly changes: SZ vs GHR + HV, SZ + GHR vs HV | • Automatic tissue and anatomical segmentation (histogram shape analysis + ANIMAL).  • Mixed model analysis, Structural Equation Modelling. | ✘ | • SZ show reduced increase in cerebral WM volume (+5.3ml) compared to GHR (+12.9 ml) or HV (+12.5 ml).  • NS between SZ+GHR vs HV. |
| Dutch Prediction of Psychosis | Ziermans, 2012 | • 8 CHR-T  • 35 CHR-NT | 30 | • Cerebral WM volume  • Voxel-wise WM density | Group differences at baseline: CHR vs HV, CHR-NT vs HV, CHR-T vs HV, CHR-NT vs CHR-T | Group effect on yearly changes: CHR vs HV, CHR-NT vs HV, CHR-T vs HV, CHR-NT vs CHR-T | • VBM analysis  • ANCOVA and linear regression.  • FDR (α=0.05, 2-tailed), post-hoc FDR (alpha=0.01). | NS | • CHR show reduced increase in cerebral WM volume compared to HV  • CHR-T show significant decrease in cerebral WM volume compared to controls  • NS changes in WM volume in CHR-NT compared to controls  • NS group effect on changes in WM density. |
|  | De Wit, 2016 | •35 UHR:  •17 Resilient  •18 Non- Resilient UHR | 24 | •Average  Volume  34 cortical structures in each hemisphere + cerebellum. | ✘ |  | •FreeSurfer 5.1.0  •Linear mixed model; relationship between age, group and brain measures. | ✘ | Resilient vs non-resilient UHR, effect of group: Resilient individuals had larger corpus callosum volume.  HV trajectories were between that of resilient and non-resilient UHR |
| Edinburgh Study of Comorbidity | Moorhead 2009 | • 53 PE | 45 | TBM maps of cerebral WM changes | ✘ | Group effect on WM changes: PE vs HV | • VBM analysis followed by SVC (left temporal, left amygdala/hippocampus)  • GLM | ✘ | • No significant effects |
| Ludwig-Maximilians | Koutsouleris 2010 | • 12 CHR-T  • 13 CHR-NT | 28 | Morphometric changes in segmented GM and WM volumes. | ✘ | Patterns of structural brain changes between time-points: CHR vs HV, CHR-T vs CHR-NT | • Deformation-based morphometry  • PLS analysis (5000 non- parametric permutation tests at α=0.05 significant level) | ✘ | • 1) CHR vs HV: WM abnormalities in CC.  (GM abnormalities in Right LPC, perisylvian, cingulate, occipital and cerebellar cortices, bilaterally)  • 2) Abnormalities more pronounced in CHR-T vs CHR-NT in CC, medial parietal cortex, MCC, PCC and periventricular structures |
| PACE | Walterfang 2008 | • 10 CHR-T  • 11 CHR-NT | ✘ | WM tissue density maps from proton density-weighted MRI | Group differences in WM volume at baseline: CHR-T vs CHR-NT | Within-group changes in WM volume in CHR-T and in CHR-NT | • ANCOVA + repeated-measures ANCOVA (1 per group)  • Cluster-level inference (cluster-forming threshold β>2, cluster mass, FWE p<0.01) | • CHR-T > CHR-NT in WM subjacent to a) left premotor cortex (SFOF*) and b) left frontal operculum (SLF)  • CHR-T < CHR-NT in a) posterior cerebellum WM and b) optic radiation. | • CHR-T: reduction in Left-FOF. Reduction in occipital lobe, subjacent to L calcarine cortex; increase in L+R posterior cerebellum.  • CHR-NT: increase in Left posterior cerebellum and WM inferior to right parietal lobe (SLF). |
| NIMH COS study | Gogtay 2012 | • 49 GHR | 57 | • WM volume  • WM growth rate | • Group differences in WM volume at baseline  • Group differences in WM growth rate at each age group | Effect of Age, GHR status, Age and GHR×Age in WM growth rate. | • Cross-sectional split in three age groups: 7 to <14 years, 14 to <18 and 18 to 28 years.  • TBM  • GLM  • FDR (p<0.05) | • WM volume: NS  • WM growth rate: in 7 to 14 years, slower Parietal WM growth rates than HV; NS in the other two groups | • Effect of Age in WM growth in HV: whole brain, parietal, frontal, occipital and temporal lobes.  • Effect of GHR status on WM growth: NS  • Effect of GHR×Age: WM growth slows down faster in HV compare to GHR in Parietal and Occipital |
| LBC1936 birth cohort (Scottish Mental Survey of 1947) | Alloza 2018 | • 488 szPGRS | ✘ | • WM tracts averages of FA and MD.  • Network connectivity measures from structural connectome | SEM: Association between MRI outcomes and szPGRS. | SEM: Association between changes in MRI outcomes and szPGRS. | • Tractometry and connectomic measures based on probabilistic tractography.  • One SEM for each tract’s FA/MD and each global graph theory measure.  • FDR (p<0.05) | NS | • Positive correlation between szPGRS and longitudinal changes in MD in splenium, AF, ATR and cingulum.  • No significant correlation between FA or graph theory metrics and szPGRS. |
| Toho University | Katagiri 2018 | • 5 CHR-T  • 23 CHR-NT-AP  • 14 CHR-NT-NAP | 16 | Volume of 5 CC cross-sectional sub-regions | Group differences in cross-sectional volumes: CHR-T vs CHR-NT vs HV | • Effect of time, medication and medication×time interaction in MRI measures in CHR-NT  • Correlation between ΔPOS and ΔNEG symptoms, and ΔROI volume | • CC subregions: (posterior, mid-posterior, central, mid-anterior and anterior)  • Processing and statistical analysis in FreeSurfer 5.2  • ANOVA and RM-ANOVA followed by Tukey's post-hoc test  • Multiple Correlation analysis | CHR-NT vs HV: Smaller volume in mid-posterior CC, central CC and mid-anterior CC | • In CHR-NT: Significant effect of time on volume mid-posterior CC (reduction), but no effect of medication or medication×time interaction.  • In CHR-NT: Improvement in negative symptoms correlated with increased volume of central CC at follow-up.  • No longitudinal data for CHR-T or HV |
| Toho University | Saito 2017 | • 7 CHR-T  • 39 CHR-NT | 16 | • WM tracts FA averages from segmented WM tracts | • Group differences at baseline: CHR vs HV, CHR-NT vs CHR-T  • Group differences at follow-up: CHR-NT vs CHR-T | Correlation between ΔPOS, ΔNEG symptoms and Tract FA averages | • DTI deterministic tractography seeding from 3 manually delineated CC ROIs following the Hofer and Frahm’s schema.  • Segmented Tracts: corpus genu, trunk and splenium of callosum.  • Group differences: Mann–Whitney U-test  • Correlations: Spearman rank order | • Baseline: FA lower in CHR than HV for all sections of CC  • Baseline: FA higher in CHR-T than CHR-NT in genu and trunk of CC.  • Follow up, FA higher in CHR-T than CHR-NT in trunk of CC | • Increase in FA over 1 year correlates with improvement of negative symptoms |
| Toho University | Katagiri 2015 | • 41 CHR:  • 7 CHR-T  • 23 CHR-NT-AP  • 11 CHR-NT-NAP | 16 | • Voxel-wise FA in TBSS skeleton  • ROI FA averages from selected TBSS ROIs. | Group differences in ROI FA averages: HV, CHR-T, CHR-NT-AP and CHR-NT-NAP | • Effect of time, medication and medication×time interaction in MRI measures in CHR-T, CHR-NT-AP and CHR-NT-NAP (RM-ANOVA)  • Correlation between ΔPOS symptoms and ROI FA averages in CHR subgroups | • TBSS analysis with Threshold-free cluster-enhancement (TFCE)  • TBSS ROIs: cluster of skeleton voxels with significant lower FA in CHR compared to HV: genu and body of the CC (left).  • ROI analysis: ANOVA followed by Tukey's post-hoc test  • Multiple Correlation analysis | Significant lower FA in CHR-NT compared to HV in a TBSS ROI with voxels in the genu and body of CC (left). | • No significant effect of time, group×time interaction or main group effect on ROI FA average.  • Improvement in sub-threshold positive symptoms correlates with increased FA in a TBSS ROI with voxels genu and body of the corpus callosum (left) |
| CHR research project, Copenhagen, Denmark | Krakauer 2018 | • 30 CHR | • 45 BL  • 23 FU | Whole-brain FA maps of TBSS skeleton | • Group differences: CHR vs HV  • Correlation between baseline FA and positive/negative symptoms at follow up. | • Effect of group and group×time interaction in MRI outcomes (GLM)  • Effect of time for each separated group  • Correlation between FA changes in positive/negative symptoms s in CHR | • TBSS with Threshold-free cluster-enhancement  • GLM, FWE (p<0.05) | • At baseline, CHR show lower FA in left CST, right ATR & left SLF.  • No significant correlations between baseline FA and positive/negative symptoms (although no one transitioned) | • No effect of group or group×time interaction.  • In CHR, significant FA increase in the left SLF which was also correlated with age.  • In HV, a significant FA increase in the left UF (no correlation with age).  • In CHR, reduction in FA (in Left IFOF, anterior thalamic radiation and SLF) correlates with a reduction of negative symptoms. |
| ADAPT Program, Northwestern University | Bernard 2015 | • 26 CHR | 21 | Tract FA averages: L-R hippocampal-thalamic white matter tracts. | Effect of group (CHR vs HV) in tract FA | • Effect of time and group×time interaction in tract FA in CHR vs HV  • Correlation between baseline FA and symptom severity after 12 months in CHR | • Probabilistic Tractography seeding from the thalamus and selecting streamlines going through the hippocampal formation.  • RM-ANCOVA  • Hierarchical regression analysis | No significant main group effect, but FA in CHR was higher than in HV at Baseline and normalised at follow-up. | • Significant group×time interaction. In HV, longitudinal FA increase in L+R hippocampal-thalamic tracts. In CHR, FA did not change in R, and slightly reduced in L.  • Increased baseline FA was associated with higher positive symptoms in CHR after 1 year. No significant correlation between baseline FA and changes in negative symptoms. |
| ADAPT Program, Northwestern University | Mittal 2014 | • 15 CHR | 15 | ROI FA averages: L-R superior cerebellar peduncles from TBSS FA skeleton. | Between group differences (CHR vs HV) in SCP FA averages at baseline | • Effect of time and group×time interaction in ROI FA averages (repeated-measures ANCOVA)  • Correlation between Neurological Soft Signs at baseline and SCP FA after 12 months in CHR | • ANCOVA and RM-ANCOVA  • Hierarchical regression analysis | No significant group differences in SCP FA averages at baseline. | • Significant group×time interaction: After 12 months, L-R SCP FA increases for HV and decreases for CHR.  • Baseline NSS correlates negatively with L-R SCP FA after 12 months (both hemispheres combined). |
| ADAPT Program, Northwestern University | Bernard 2017 | • 26 CHR | 24 | Tract FA averages for Cerebello-thalamo-cortical WM tract segments | ✘ | • Longitudinal changes (paired t-test in CHR group alone).  • Effect of group×time interaction in Tract FA averages (2x2 ANOVA)  • Correlations between FA changes and positive/negative symptoms in CHR. | • Cerebello-thalamo-cortical WM tract segments: 1) Lobule V-thalamic, 2) Crus I-thalamic, 3) thalamo-prefrontal, 4) thalamo-motor  • Bonferroni correction for ANOVA: for 4 tract segments (p < 0.0125)  • Bonferroni correction correlations: for 4 tract segments and positive/negative (p < 0.006). | ✘ | • Significant group×time: In CHR FA decreased in 3 out of 4 tract segments (not thalamo-motor), whereas FA increased in HV.  • Positive correlation between FA change and positive symptom change in thalamo-motor Tract: increased FA correlates with worsening symptoms over time.  • Also, quadratic correlation: those with biggest reduction/increase in FA had worst outcome |
| Genetic Risk and Outcome of Psychosis, Netherlands | Domen 2017 | • 55 GHR siblings  • 55 psychotic patients | 49 | ROI FA averages from 19×2 regions | Group differences in whole-brain FA at follow-up | • Longitudinal changes (within-group paired t-test)  • Effects of group, group×sex and group×region in regional ΔFA | • 19×2 regions from (JHU ICBM)-DTI-81 WM atlas intersected with TBSS FA skeleton  • Mixed effects model  • Simes’ procedure used to control the false discovery rate (p< 0.05). | • Patients and siblings showed significant lower whole-brain FA than controls at follow-up.  • Patients-sibling whole-brain FA comparison was neither large nor significant at follow-up. | • In GHR siblings compared to controls, significant decrease in whole-brain FA.  • No significant effect of group or group×sex in regional ΔFA.  • Significant effect of group×region in ΔFA: compared to controls, patients had a significant smaller ΔFA increase in R retrolenticular part of internal capsule, and a decrease in FA in R Posterior corona radiata and R cingulum. Compared to controls, siblings had a decrease in FA in the Left-PTR. |
| OASIS, London | Carletti 2012 | • 15 FEP  • 22 CHR:  • 5 CHR-T  • 17 CHR-NT | 32 | Whole-brain maps of DTI outcomes: FA, RD, AD. | Between-group differences: CHR vs FEP vs HV | Effect of group×time interaction | • ANOVA and non-parametric RM-ANOVA  • Cluster-level inference (cluster mass)  • Multiple comparisons: cluster mass threshold (expected # type I error clusters < 1) | • Baseline FA: FEP < CHR < HV. Clusters: 1) splenium and body of CC, L IFL and SLF, L IFOF. 2) R external capsule, retrolenticular part of R internal capsule, and R posterior corona radiata  • Baseline RD: FEP < CHR < HV. Larger number of clusters across white matter.  • Baseline AD: Many clusters with either FEP < CHR < HV or FEP > CHR > HV  • All between-group differences driven by FEP vs HV as CHR vs HV was not significant.  • Baseline CHR-T vs CHR-NT: Non-significant.  • Baseline CHR-T vs HV: Non-significant  • Follow-up CHR-T vs CHR-NT: FA Non-significant, RD goes up in MCP, AD goes up in R Sup and Post Cor Rad, splenium and body CC, AD goes down in MCP and LCP. | • At follow up, FA goes down for CHR-T while it slightly increases for CHR-NT in a cluster encompassing the anterior limb of the left internal capsule (ALIC), body of the corpus callosum, left superior corona radiata, and left SFOF. |
| Philadelphia Neurodevelopmental Cohort | Roalf 2019 | • BL: 38 PE  • FU: 37 PE | • BL: 79  • FU: 89 | • Whole-brain average and voxel-wise FA, MD, AD, and RD across TBSS WM skeleton.  • ROI diffusion metrics averages were extracted from 10 ROIs. | • Group differences: PE vs HV  • Mixed-effects models (include participants with 1 timepoint). Diagnosis x age  • Association of MR outcomes with clinical symptoms and cognitive performance | Similarity of diffusion metrics over time (ICC) | • All participants with at least 1 timepoint included in the analysis.  • TBSS analysis: non-linear implementation of FSL randomise with TFCE and FWE p<0.05.  • Generalized additive mixed model  • ROI analysis GAMM implementation in R with FDR (q-value=0.05).  • ICC analysis in R with I2C2 | • Whole-brain FA: PE < HV  • TBSS FA: Significant differences in forceps major, inferior frontal occipital fasciculus (IFOF), cingulate gyrus proximal to the hippocampus (CGH), corticospinal tracts (CST), ATR, ILF, SLF.  • ROI FA: PE < HV in CST, CGH.  • Whole-brain RD: PE > HV  • TBSS RD: Sign differences in forceps major, IFOF.  • ROI RD: Non-Significant  • MD and AD: No significant differences for Whole-brain, TBSS and ROI measures.  • Positive symptoms associated with higher whole-brain averages (MD, AD, RD), ILF averages (MD, AD, RD) and IFOF averages (MD, RD).  • Worse cognitive performance associated with higher whole-brain averages (MD, AD), CST and UF averages (AD) and CGH averages (RD). | • In general, FA values had higher ICCs than measures of diffusivity.  • Differences between ROI ICC values for PE and HV went from 0 to 0.18.  • ROIs were ICC differences between PE and HV were > 0.1: CST (MD, AD, RD), CGC (MD), CGH (AD, RD), IFO (MD) and SLF (FA, MD, RD). |

### Results by research group

The Edinburgh High-Risk Study examined subjects at genetic high risk of schizophrenia (GHR), aged between 16 and 25 years with at least two affected relatives. A number of structural neuroimaging analysis pipelines have been applied to the Edinburgh High-Risk Study. GHR participants showed progressive grey matter volume loss in bilateral temporal, right frontal and right parietal lobes (1) although there were no group differences with HV. In a larger overlapping sample, group differences between GHR and HV emerged, with greater reductions in whole brain, left and right prefrontal and temporal lobe (2). This was paralleled by cortical thinning in the frontal, cingulate, and occipital lobes, but preserved surface area in GHR, whilst in HV cortical thickness increased and surface area decreased over time (3). Group differences were also detected in an ROI study of the hippocampus, where volume did not change over time in GHR but increased in HV (4). Temporal lobe volume changes were not detected in another study using semi-automated hand tracing of ROIs (5).

When clinical status was examined in the Edinburgh High-Risk Study, right temporal lobe volume reduced over time in symptomatic GHR in comparison to non-symptomatic GHR (5). In those who transitioned to psychosis, prefrontal volume reduced over time in comparison to symptomatic and non-symptomatic GHR (2). In contrast, two ROI studies of the amygdala-hippocampus found no group differences based on clinical presentation (1,4), although grey matter loss was more widespread in those who developed schizophrenia (1), and reduction in the inferior temporal gyrus provided a 60% positive predictive value for developing schizophrenia (6). For cortical thickness, no differences were found between clinical subgroups (ill, symptomatic and well) (3).

The Edinburgh Study of Comorbidity is a separate cohort which examined adolescents with cognitive impairment. In those with schizotypal features (classified as PE in this review), there was significantly greater loss in temporal lobe grey matter compared to those without PE (Moorhead et al., 2009), and worsening symptoms correlated with grey matter loss. No differences were seen in white matter volume. Further study of those with schizotypal features reported greater grey matter loss in individuals with prominent negative symptoms compared to those without, in left posterior cingulate, cerebellum, temporal lobe and occipital lobe (McKechanie et al., 2016). In summary, in the Edinburgh studies, differences in brain trajectory appear to be strongest between GHR and HV subjects in the frontal and temporal lobes, which can be more pronounced in those who remain symptomatic.

The IMAGEN project is a European multicentre neuroimaging study across 8 research centres. Higher scores on the Community Assessment of Psychic Experiences (CAPE) were related to a smaller expansion of the right uncus in the parahippocampus formation between the ages of 14 to 19 years (7). Moreover, the deficit in uncus development partially mediated the association between cannabis use and psychotic-like experiences.

In the US Pittsburgh cohort of first and second-degree relatives of schizophrenia and schizoaffective patients, an ROI study of the superior temporal gyrus found reductions in surface area, specific to GHR males in the left hemisphere (8). Further study of a subset of this sample revealed a decline in grey matter volume in GHR, whereas grey matter in HV remained stable for orbitofrontal, medial prefrontal, anterior cingulate, and increased in temporal pole and right inferior frontal gyrus (9). The exception was the left inferior gyrus, which remained stable in GHR but reduced in HV. Furthermore, steeper longitudinal reductions predicted worsening of symptoms over time. Although grey matter progressively decreased in GHR in this cohort, differences were also present at baseline, with smaller amygdalae, frontal and parietal gyri in GHR compared to HV. Moreover smaller volumes at baseline predicted greater prodromal symptoms at follow-up. The greatest volume reduction was seen in the mPFC, and the strongest association with symptomatology occurred in the mPFC and temporal pole. Thus abnormal maturation of these brain regions during adolescence may be involved in the pathogenesis of schizophrenia, although no participants transitioned to psychosis after 1 year. A limitation of the study is that results did not survive correction for multiple comparisons.

The same research group examined offspring of patients with schizophrenia and measured cortical surface area, curvature and thickness over one year (10). A reduction in frontal and occipital surface area, alongside preserved or increased cortical thickness in temporal and parietal lobes was seen in GHR compared to HV. Cortical thinning occurs as part of normal development, and so these results may point to halted development in GHR. However, the majority of studies find greater cortical thinning in the temporal lobe of high risk groups (see the NIMH cohort below as well as Hedman et al., 2016; Ziermans et al., 2012). Regarding surface area, these results contrast with the Edinburgh High-Risk study where global surface area was preserved over time in GHR (3), and the Barcelona and Dutch Prediction of Psychosis cohorts, where smaller longitudinal decreases in cortical surface area in HR compared to healthy volunteers (13,14).

The ADAPT Program at Northwestern University, Illinois, examined local gyrification index, mean curvature index, and sulcal depth in clinical high-risk youths (15). In general, baseline measures of cortical surface morphometry were lower in CHR subjects (hypogyrification and reduced sulcal depth), particularly in the parietal lobe, which did not change over time and remained stable over 1 year. It should be noted longitudinal changes were assessed by intraclass correlation, which typically assesses the reliability of measurements. In a subset of the cohort, fractional anisotropy (FA) was examined in tracts connecting Lobule V of the cerebellum to the motor cortex in the frontal lobe, and Crus I to the prefrontal cortex (both via the thalamus) (16). The normal developmental FA increase in HV was not seen in CHR, where FA reduced in the Lobule V to thalamic tract, Crus I-thalamic and thalamo-prefrontal tract segment. This was consistent with an earlier study in a subset of the sample, wherein FA increased over time in HV but reduced in CHR in the superior cerebellar peduncle (17). In the thalamo-motor tract a quadratic relationship was found between the change in FA and the change in symptoms; CHR subjects who showed the greatest degree of change in FA (increase or decrease) exhibited worsening positive symptoms (16). An ROI study examined white matter connections between the thalamus and hippocampus (18). Again, in HV FA increased over time in both hemispheres, whereas in CHR FA did not change (right hemisphere) or slightly decreased (left hemisphere). Higher baseline FA correlated with higher positive symptoms 1 year later.

In the GROUP cohort in the Netherlands, 30 year old GHR subjects were followed up for 3 years (19). Whole brain mean FA did not differ between GHR and HV at baseline, but reduced over time in GHR and increased in HV. Region analysis showed that in GHR compared to HV, FA reduced over time in the left posterior thalamic radiation and right cingulum, and had smaller increases in the right retrolenticular part of internal capsule. Patients with psychotic disorders (mean duration of illness 5 years) had lower whole-brain mean FA values than HV at both timepoints, but their trajectories did not differ from HV. At the regional level however, FA in patients reduced over time in the right cingulum, had a higher decrease in the right posterior corona radiata and a smaller increase in the right retrolenticular part of the internal capsule.

The PACE clinic in Melbourne recruited CHR participants and compared them based on clinical outcome. In CHR participants that go on to transition (CHR-T), there was a reduction in grey matter volume over time in frontal and temporal regions, whereas in those who do not transition to psychosis (CHR-NT), reductions between baseline and follow-up were only seen in a region of left cerebellum (20). No significant interaction between group and time was found. Group differences emerged when brain surface area was studied, as CHR-T showed greater prefrontal contraction compared to CHR-NT (0.4mm contraction in brain surface area compared to 0.2mm respectively) (21). This change in the lateral cortical surface is below standard voxel resolution, and so would not have been detected using typical methods such as grey matter density. A previous study using different surface morphology methods found no difference between clinical subgroups (3), whilst other studies of surface area have focused on GHR vs HV contrasts (no group differences between GHR and HV (11,15) reduction in GHR (10), no change in GHR but reduction in HV (3) and smaller longitudinal decreases in cortical surface area in HR compared to healthy volunteers (13,14)).

A later study in the PACE cohort conducted an ROI analysis of the superior temporal gyrus volume (STG) and insula. STG and insula volume reduced over time in those who transitioned compared to both HV and CHR-NT (22,23). Baseline white matter volume was higher in the SFOF and SLF in those who went on to transition, compared to those who did not transition. Over time, CHR-T subjects showed a reduction in the left fronto-occipital fasciculus and in the white matter subjacent to the left calcarine cortex, and an increase in the posterior cerebellum, whereas CHR-NT subjects showed no reductions in white matter, only an increase in the left posterior cerebellum and in a white-matter region subjacent to the right inferior parietal lobule close to the SLF (24). In summary, the findings from the PACE cohort indicate that the time preceding transition is associated with an accelerated decline in grey matter and surface area in frontal and temporal regions, alongside altered white matter development in the frontal and occipital lobes. One study in this cohort also imaged first-episode psychosis patients, where extensive volume reductions in STG were already present at baseline. This indicates that volume loss precedes florid psychosis, and progresses further with disease.

The FEPSY project, Switzerland, examined CHR participants and found reductions in temporal lobe, as well as frontal, parietal and cerebellum grey matter volume, only in those who transitioned to psychosis, whereas no changes were seen in those who did not transition. Between-group differences were not examined, and a control group was not included (25). An ROI analysis in the hippocampus and pituitary gland found no significant change over time in grey matter between CHR-T vs CHR-NT (26,27). The Singapore Longitudinal Youth At-Risk Study in CHR participants also examined an ROI in the hippocampus (28). A reduction in CA1 volume was detected in persistently symptomatic CHR individuals compared to those whose symptoms remit and HV, and this decline was associated with worsening symptoms over time. No longitudinal differences were found between remitted participants and HV, and there were no baseline differences between subgroups.

The OASIS cohort recruited help-seeking individuals in London who met criteria for an At-Risk Mental State (29). Grey matter volume did not change over 2 years in HV, whereas in CHR, there was reduced volume in the superior frontal gyrus bilaterally and right caudate, and increases in the inferior frontal gyrus, anterior cingulate and cerebellum. Longitudinal differences between groups were not examined. Participants that later transitioned to psychosis showed grey matter loss in the right middle temporal gyrus, whereas those who did not transition showed grey matter volume loss in the superior frontal gyrus bilaterally, right caudate and left putamen, with increases in the inferior frontal gyrus bilaterally and right anterior cingulate. Volume differences between CHR and HV were present at baseline, with smaller right temporal gyri, superior frontal and orbital gyrus, and left medial and inferior frontal gyrus in CHR participants. Furthermore, grey matter volume in the right inferior parietal lobule and left parahippocampal gyrus was smaller at baseline in those who went on to transition compared to CHR-NT. Differences in white matter FA values were not found between CHR-T and CHR-NT at baseline, however fractional anisotropy was lowest in first episode psychosis patients, highest in controls, and intermediate in CHR subjects (30). Over time a progressive reduction in FA of the left frontal white matter was found in CHR subjects who went on to develop psychosis compared to a slight increase in CHR subjects who did not transition (in a cluster spanning the left ALIC, body of the corpus callosum, left superior corona radiata, and left SFOF).

At Toho University, Japan, in a study of CHR participants who did not develop psychosis, putamen and mid-posterior corpus callosum volume reduced over time, irrespective of antipsychotic medication status (31,32), but trajectories in healthy volunteers or in transitioned subjects were not examined. Reduced putamen volume in CHR-NT is consistent with the OASIS cohort above. Improvement in sub-threshold positive symptoms correlated with increased volume in the right accumbens (31), and improvement in negative symptoms correlated with an increase in the volume of the central corpus callosum at follow up (32). This suggests that maturation of the putamen and corpus callosum may be protective against the development of psychosis, although comparisons with healthy volunteer trajectories are needed. In another study of the same cohort, baseline FA in the left portions of the genu and body of the corpus callosum were lower in CHR-NT compared to HV, and an improvement in positive symptoms (33) and negative symptoms (34) was associated with an increase in FA over time in those regions. At baseline FA was higher in CHR-T compared to CHR-NT in the genu and trunk of the corpus callosum, and at follow up, FA was higher in CHR-T in the trunk of the corpus callosum.

In the Copenhagen cohort, there was no interaction between group and time. Post-hoc analyses in CHR found a significant increase in FA in the left SLF over time, which was also correlated with age, and in HV a significant FA increase in the left uncinate fasciculus (no correlation with age). At baseline FA was lower in CHR compared to HV in the left corticospinal tract, right ATR and left SLF (35). The mean age of CHR subjects was 24 years at baseline, where FA values typically plateau, suggesting that CHR subjects do not reach peak white matter maturation. Reduced FA over time in the three regions as well as the left frontooccipital fasciculus correlated with improved negative symptoms.

The Philadelphia Neurodevelopmental Cohort (PNC) in the US followed up typically developing subjects over 2 years, alongside youth who presented with psychotic experiences at both timepoints (PE group). FA increased and radial diffusivity decreased over time with age across the cohort, but PE subjects showed consistently lower whole-brain and regional FA, in TBBS clusters comprising the forceps major, inferior frontal occipital fasciculus (IFOF), anterior thalamic radiate, inferior and superior longitudinal fasciculus, CGH, and the CST (36). Regional analyses found significant reductions in the latter two regions in PE compared to HV. Furthermore TBSS analyses of radial diffusivity (RD) found higher RD in a cluster comprising the IFOF and forceps major in the right hemisphere of PE subjects compared to HV, which were not significant in region analyses. Therefore, consistent with the Copenhagen cohort, high risk groups had reduced FA compared to HV which do not progress over time. This indicates that white matter development may be a trait marker of vulnerability to psychosis, rather than relating to symptom expression. Unlike the Toho University cohort (32,34), no differences in corpus callosum FA were found.

The NIMH COS study in the US followed siblings of patients with childhood-onset schizophrenia in an accelerated longitudinal design, whereby participants aged between 8 and 28 years (mean age 16 years) were invited to be scanned at 4 timepoints, each 2 years apart. The majority of subjects received 2 MRI scans. GHR participants showed early cortical thinning in prefrontal, superior temporal and inferior parietal cortex compared to HV (37). HV showed steeper rates of cortical thinning with age, eventually merging with GHR trajectories so that no abnormalities remained by age 20 years. In child-onset schizophrenia, grey matter loss did not normalise in prefrontal and temporal lobes, and early grey matter loss in the parietal lobe was more widespread than the circumscribed region seen in GHR (38). These results suggest that grey matter development in GHR is shifted to earlier ages. The normalisation of GM deficits over time may be protective in GHR, although flatter trajectories of cortical thinning may impact intelligence, as delayed cortical development followed by a steep trajectory of frontal cortical thinning is associated with high intelligence (39). Taken together, deficits in frontal and temporal lobe maturation may represent genetic liability to disease, and a further progression of these deficits may lead to psychosis. A replication study in a non-overlapping, slightly younger cohort (mean age 13) confirmed these findings, reporting early prefrontal, temporal and parietal deficits in GHR, that normalized by late adolescence (40).

An extension of the original NIMH study included a further 34 siblings and 39 patients (41), which examined structural connectivity between pairs of cortical regions. In GHR, connectivity between the left temporal lobe and left occipital cortex was reduced in comparison to HV, but normalised by age 17. Patients with childhood-onset schizophrenia showed significantly longer maturational delays, with connectivity between these regions not developing until early adulthood. These changes likely reflect genetic vulnerability which may lead to illness if deficits proceed beyond a critical time frame. White matter tracts have not been examined in this cohort, and it would be of interest whether development of the inferior longitudinal fasciculus (ILF) is altered, as this tract connects the occipital lobe with the anterior part of the temporal lobe, where altered structural connectivity was detected. White matter volume growth was delayed in GHR in the parietal lobe, which normalised in late adolescence (42). Finally, ROI studies of brain volume in this cohort reveal a reduction in cerebellum (43) but not hippocampus volume (44) in GHR subjects compared to HV. Although no significant differences were detected in hippocampus, the greatest volume deviation from HV was seen in the youngest participants (aged 12), which normalised over time. In summary, deficits in cortical thickness, structural connectivity and white matter development are present in GHR participants at a young age, but normalise by early adulthood.

In a cohort of PE subjects sampled from Dublin and Kildare, hippocampal volume was lower at both timepoints, at 13 and 15 years of age, compared to healthy volunteers (45). Baseline MRI measures were taken at an age similar to the NIMH cohort, but were followed up after 2 years rather than 6-10 years. It would be of interest to see whether these volume differences normalise at a later follow-up.

The twin pair cohort at the University Medical Centre Utrecht examined monozygotic (MZ) and dizygotic (DZ) twin pairs discordant for schizophrenia. In healthy co-twins of patients there was a progressive global thinning of the cortex, particularly in the left superior temporal cortex, in comparison to HV (11). Excessive thinning of prefrontal and parietal cortices was present but did not survive correction for multiple comparisons (left inferior parietal and angular cortices, as well as the left frontal middle and right superior orbitofrontal cortices). Cortical surface area was not related to genetic liability for schizophrenia, which contrasts with Prasad et al., 2010, who studied younger offspring of patients with schizophrenia, rather than their adult co-twins. In the Utrecht sibling-pair cohort, there were no differences in brain volume over time between GHR and HV (46), but schizophrenia patients showed smaller increases in cerebral WM volume and reduced cerebral grey matter volume than GHR and HV. Altered brain trajectories may be present in co-twins but not siblings due to the higher genetic risk load in twins. GHR subjects were on average 40 years old, and as suggested by studies in the NIMH cohort, abnormal brain trajectories may have normalised by this time in siblings.

The Dutch Prediction of Psychosis Study measured total brain, grey and white matter volumes and cortical thickness, and found delayed development of cerebral WM volume (smaller increases in white matter volume compared to HV) and increased cortical thinning in the left middle temporal gyrus over time in CHR (12). More prominent changes over time were seen in those who transitioned, with a greater loss in total brain grey matter volume compared to non-transition participants and HV, and a reduction in white matter volume in contrast to increased volume in HV and progressive cortical thinning in the left anterior cingulate cortex, precuneus, and parts of the temporo-parieto-occipital area compared with HV. Grey matter trajectories significantly differed between CHR-NT and CHR-T, as changes in CHR-NT were similar to HV, whereas white matter trajectories did not significantly differ between CHR-NT and CHR-T, as changes were intermediate in CHR-NT between that of HV and CHR-T.

A longer term follow-up of 6 years in the Dutch Prediction of Psychosis found the largest differences in surface area, with less steep decreases over time in frontal and parietal areas in UHR compared to HV (47). Over both time points UHR participants had smaller hippocampus and thalamic volumes, which also decreased further over time in UHR, and larger third and inferior lateral ventricles which increased over time. This study split UHR subjects into resilient and non-resilient groups based upon outcomes at 6 years. Differences in brain volume and cortical thickness that were already present at baseline were stable over development, with resilient individuals showing larger volumes of frontal, temporal and parietal cortex, corpus callosum and nucleus accumbens than non-resilient individuals. When compared with healthy volunteers, the trajectories of volume and thickness were parallel and highest in the resilient group, intermediate in HV and lowest in non-resilient. Similarly, mean brain cortical thickness was larger for resilient compared to non-resilient individuals, due to increased thickness in frontal, parietal and temporal lobes. The white matter results differ from Ziermans et al., described above, which examines cerebral WM volume over 2 years in the same cohort, where healthy volunteers had the highest white matter volume (12). The 6-year study also identified interactions between group and time in certain brain regions; finding larger decreases in volume over time in anterior cingulate gyrus and a larger increase in lateral ventricle volume in non-resilient individuals, consistent with Ziermans et al., alongside decreased surface area over time in precentral gyrus and frontal pole in non-resilient UHR compared to increases in resilient UHR. Cortical thickness was lower in non-resilient UHR at baseline in superior temporal cortex and posterior cingulate gyrus, and both resilient and non-resilient individuals showed decreases over time in cortical thickness in these areas, however, the non-resilient group showed a slower rate of change than the resilient group, in contrast to Ziermans et al., alongside decreases in gyrification in anterior cingulate gyrus over time in resilient UHR compared to increases in non-resilient UHR.

At Ludwig-Maximilians-University, Germany, a multivariate approach was applied to structural MRI data in CHR participants. Morphometric abnormalities in higher-order cortical networks and subcortical structures were found in CHR compared to HV, in the corpus callosum, right prefrontal, as well as the perisylvian (including insular cortex and superior temporal gyri), cingulate, parietal, occipital, caudate nucleus and periventricular structures (48). These abnormalities were more pronounced in CHR-T versus CHR-NT, in the corpus callosum, medial parietal cortex, middle and posterior cingulate cortex and periventricular structures. Moreover, baseline scans were able to predict the longitudinal volumetric changes of each CHR subgroup.

The US NAPLS longitudinal multicentre study consists of the largest sample to date of individuals at clinical high risk (n=274). In CHR participants that transitioned to psychosis, greater cortical thinning in the frontal cortex and greater expansion of the third ventricle was evident in comparison to non-transition subjects and HV (49). This is consistent with reduced frontal volume reported in Borgwardt et al., 2008, Mcintosh et al., 2011, and Pantelis et al., 2003. Importantly, the study showed that cortical thinning was not the result of antipsychotic exposure, as analyses remained significant when restricted to subjects without exposure to antipsychotic medication. Grey matter loss was most pronounced in converters with a short duration of prodromal symptoms.

Furthermore, in the CHR-T group, unusual thought content at baseline associated with steeper grey matter decline in the middle frontal gyrus and expansion in the third ventricle (Chung et al., 2015). No association with symptom severity was found in CHR-NT. More widespread cortical thinning may be apparent in CHR-T, as ventricular expansion was associated with a progressive reduction of grey matter in PFC, superior temporal gyrus, and parietal cortices (Chung et al., 2017). In the original analysis, a reduction in cortical thickness was observed in the right superior and inferior parietal cortex, superior temporal gyrus, and parahippocampal gyrus compared with control subjects, however this did not survive FDR-correction, with only the frontal clusters remaining significant.

A study of a Scottish healthy older age birth cohort assessed the association between polygenic risk score for schizophrenia (szPGRS) and DTI measures (50). Greater genetic risk for schizophrenia was associated with longitudinal increases in mean diffusivity (MD) in the splenium, arcuate, ATR and cingulum fasciculi over 3 years, but no association with change in fractional anisotropy (FA) or structural brain connectivity. The cohort was on average 76 years of age, and so age-related degeneration of white matter tracts appears to be greater in those with higher genetic risk.

In Barcelona, offspring of patients with schizophrenia or bipolar disorder were recruited at a young age (6 to 17 years old) and were followed up after 2 and 4 years. The schizophrenia offspring group exhibited smaller global, parietal, and occipital lobe surface area at baseline compared with HV, but no differences in cortical thickness. Offspring were split into pre-pubertal and pubertal groups, and observed a smaller longitudinal decrease in cortical surface area and thickness in pubertal schizophrenia offspring compared to HV and bipolar offspring respectively, over 2 years (13). When all the timepoints were assessed, bipolar and schizophrenia offspring were combined into one group (=GHR), and post-hoc tests examined bipolar and schizophrenia offspring separately. At baseline, GHR participants who developed psychotic spectrum symptoms had smaller total surface area and grey matter volume than those who did not develop symptoms and controls. GHR participants who developed psychotic spectrum symptoms showed greater time-related mean cortical thinning than those who did not develop symptoms and controls. This effect was present in both schizophrenia and bipolar disorder offspring in the occipital cortex. GHR showed smaller longitudinal decreases in surface area than controls, which was driven by schizophrenia offspring who did not develop symptoms (51), whereas symptomatic GHR had smaller mean SA at all timepoints. Similarly, non-symptomatic schizophrenia offspring showed smaller decreases in total grey matter volume than HV.

### References

1. Job DE, Whalley HC, Johnstone EC, Lawrie SM. Grey matter changes over time in high risk subjects developing schizophrenia. *Neuroimage* (2005) **25**:1023–1030. doi:10.1016/j.neuroimage.2005.01.006

2. Mcintosh AM, Owens DC, Moorhead WJ, Whalley HC, Stanfield AC, Hall J, Johnstone EC, Lawrie SM. Longitudinal volume reductions in people at high genetic risk of schizophrenia as they develop psychosis. *Biol Psychiatry* (2011) **69**:953–958. doi:10.1016/j.biopsych.2010.11.003

3. Bois C, Ronan L, Levita L, Whalley HC, Giles S, McIntosh AM, Fletcher PC, Owens DC, Johnstone EC, Lawrie SM. Cortical Surface Area Differentiates Familial High Risk Individuals Who Go on to Develop Schizophrenia. *Biol Psychiatry* (2015) **78**:413–420. doi:10.1016/j.biopsych.2014.12.030

4. Bois C, Levita L, Ripp I, Owens DCG, Johnstone EC, Whalley HC, Lawrie SM. Longitudinal changes in hippocampal volume in the Edinburgh High Risk Study of Schizophrenia. *Schizophr Res* (2016) **173**:146–151. doi:10.1016/j.schres.2014.12.003

5. Lawrie SM, Whalley H, Abukmeil SS, Kestelman JN, Miller P, Best JJK, Owens DGC, Johnstone EC. Temporal lobe volume changes in people at high risk of schizophrenia with psychotic symptoms. *Br J Psychiatry* (2002) **181**:138–143. doi:10.1017/S0007125000161860

6. Job DE, Whalley HC, McIntosh AM, Owens DGC, Johnstone EC, Lawrie SM. Grey matter changes can improve the prediction of schizophrenia in subjects at high risk. *BMC Med* (2006) **4**: doi:10.1186/1741-7015-4-29

7. Yu T, Jia T, Zhu L, Desrivières S, Macare C, Bi Y, Bokde ALW, Quinlan EB, Heinz A, Ittermann B, et al. Cannabis-Associated Psychotic-like Experiences Are Mediated by Developmental Changes in the Parahippocampal Gyrus. *J Am Acad Child Adolesc Psychiatry* (2020) **59**:642–649. doi:10.1016/j.jaac.2019.05.034

8. Bhojraj TS, Sweeney JA, Prasad KM, Eack S, Rajarethinam R, Francis AN, Montrose DM, Keshavan MS. Progressive alterations of the auditory association areas in young non-psychotic offspring of schizophrenia patients. *J Psychiatr Res* (2011) **45**:205–212. doi:10.1016/j.jpsychires.2010.05.018

9. Bhojraj TS, Sweeney JA, Prasad KM, Eack SM, Francis AN, Miewald JM, Montrose DM, Keshavan MS. Gray matter loss in young relatives at risk for schizophrenia: Relation with prodromal psychopathology. *Neuroimage* (2011) **54**: doi:10.1016/j.neuroimage.2010.04.257

10. Prasad KM, Goradia D, Eack S, Rajagopalan M, Nutche J, Magge T, Rajarethinam R, Keshavan MS. Cortical surface characteristics among offspring of schizophrenia subjects. *Schizophr Res* (2010) **116**:143–151. doi:10.1016/j.schres.2009.11.003

11. Hedman AM, van Haren NEM, van Baal GCM, Brouwer RM, Brans RGH, Schnack HG, Kahn RS, Hulshoff Pol HE. Heritability of cortical thickness changes over time in twin pairs discordant for schizophrenia. *Schizophr Res* (2016) **173**:192–199. doi:10.1016/j.schres.2015.06.021

12. Ziermans TB, Schothorst PF, Schnack HG, Koolschijn CMPP, Kahn RS, van Engeland H, Durston S. Progressive Structural Brain Changes During Development of Psychosis. *Schizophr Bull* (2012) **38**:519–530. doi:10.1093/schbul/sbq113

13. Sugranyes G, Solé-Padullés C, de la Serna E, Borras R, Romero S, Sanchez-Gistau V, Garcia-Rizo C, Goikolea JM, Bargallo N, Moreno D, et al. Cortical Morphology Characteristics of Young Offspring of Patients With Schizophrenia or Bipolar Disorder. *J Am Acad Child Adolesc Psychiatry* (2017) **56**:79–88. doi:10.1016/j.jaac.2016.09.516

14. de Wit S, Wierenga LM, Oranje B, Ziermans TB, Schothorst PF, van Engeland H, Kahn RS, Durston S. Brain development in adolescents at ultra-high risk for psychosis: Longitudinal changes related to resilience. *NeuroImage Clin* (2016) **12**:542–549. doi:10.1016/j.nicl.2016.08.013

15. Damme KSF, Gupta T, Nusslock R, Bernard JA, Orr JM, Mittal VA. Cortical Morphometry in the Psychosis Risk Period: A Comprehensive Perspective of Surface Features. *Biol Psychiatry Cogn Neurosci Neuroimaging* (2019) **4**:434–443. doi:10.1016/j.bpsc.2018.01.003

16. Bernard JA, Orr JM, Mittal VA. Cerebello-thalamo-cortical networks predict positive symptom progression in individuals at ultra-high risk for psychosis. *NeuroImage Clin* (2017) **14**:622–628. doi:10.1016/j.nicl.2017.03.001

17. Mittal VA, Dean DJ, Bernard JA, Orr JM, Pelletier-Baldelli A, Carol EE, Gupta T, Turner J, Leopold DR, Robustelli BL, et al. Neurological soft signs predict abnormal cerebellar-thalamic tract development and negative symptoms in adolescents at high risk for psychosis: A longitudinal perspective. *Schizophr Bull* (2014) **40**:1204–1215. doi:10.1093/schbul/sbt199

18. Bernard JA, Orr JM, Mittal VA. Abnormal hippocampal-thalamic white matter tract development and positive symptom course in individuals at ultra-high risk for psychosis. *npj Schizophr* (2015) **1**: doi:10.1038/npjschz.2015.9

19. Domen P, Peeters S, Michielse S, Gronenschild E, Viechtbauer W, Roebroeck A, Van Os J, Marcelis M. Differential time course of microstructural white matter in patients with psychotic disorder and individuals at risk: A 3-year follow-up study. *Schizophr Bull* (2017) **43**:160–170. doi:10.1093/schbul/sbw061

20. Pantelis C, Velakoulis D, McGorry PD, Wood SJ, Suckling J, Phillips LJ, Yung AR, Bullmore ET, Brewer W, Soulsby B, et al. Neuroanatomical abnormalities before and after onset of psychosis: a cross-sectional and longitudinal MRI comparison. *Lancet* (2003) **361**:281–288. doi:10.1016/S0140-6736(03)12323-9

21. Sun D, Phillips L, Velakoulis D, Yung A, McGorry PD, Wood SJ, van Erp TGM, Thompson PM, Toga AW, Cannon TD, et al. Progressive brain structural changes mapped as psychosis develops in “at risk” individuals. *Schizophr Res* (2009) **108**:85–92. doi:10.1016/j.schres.2008.11.026

22. Takahashi T, Wood SJ, Yung AR, Soulsby B, McGorry PD, Suzuki M, Kawasaki Y, Phillips LJ, Velakoulis D, Pantelis C. Progressive gray matter reduction of the superior temporal gyrus during transition to psychosis. *Arch Gen Psychiatry* (2009) **66**:366–376. doi:10.1001/archgenpsychiatry.2009.12

23. Takahashi T, Wood SJ, Yung AR, Phillips LJ, Soulsby B, McGorry PD, Tanino R, Zhou SY, Suzuki M, Velakoulis D, et al. Insular cortex gray matter changes in individuals at ultra-high-risk of developing psychosis. *Schizophr Res* (2009) **111**:94–102. doi:10.1016/j.schres.2009.03.024

24. Walterfang M, McGuire PK, Yung AR, Phillips LJ, Velakoulis D, Wood SJ, Suckling J, Bullmore ET, Brewer W, Soulsby B, et al. White matter volume changes in people who develop psychosis. *Br J Psychiatry* (2008) **193**:210–215. doi:10.1192/bjp.bp.107.043463

25. Borgwardt SJ, McGuire PK, Aston J, Gschwandtner U, Pflüger MO, Stieglitz RD, Radue EW, Riecher-Rössler A. Reductions in frontal, temporal and parietal volume associated with the onset of psychosis. *Schizophr Res* (2008) **106**:108–114. doi:10.1016/j.schres.2008.08.007

26. Walter A, Studerus E, Smieskova R, Kuster P, Aston J, Lang UE, Radue EW, Riecher-Rössler A, Borgwardt S. Hippocampal volume in subjects at high risk of psychosis: A longitudinal MRI study. *Schizophr Res* (2012) **142**:217–222. doi:10.1016/j.schres.2012.10.013

27. Walter A, Studerus E, Smieskova R, Tamagni C, Rapp C, Borgwardt SJ, Riecher-Rössler A. Pituitary gland volume in at-risk mental state for psychosis: A longitudinal MRI analysis. *CNS Spectr* (2015) **20**:122–129. doi:10.1017/S109285291400011X

28. Ho NF, Holt DJ, Cheung M, Iglesias JE, Goh A, Wang M, Lim JK, De Souza J, Poh JS, See YM, et al. Progressive Decline in Hippocampal CA1 Volume in Individuals at Ultra-High-Risk for Psychosis Who Do Not Remit: Findings from the Longitudinal Youth at Risk Study. *Neuropsychopharmacology* (2017) **42**:1361–1370. doi:10.1038/npp.2017.5

29. Fusar-Poli P, Crossley N, Woolley J, Carletti F, Perez-Iglesias R, Broome M, Johns L, Tabraham P, Bramon E, McGuire P. Gray matter alterations related to P300 abnormalities in subjects at high risk for psychosis: Longitudinal MRI-EEG study. *Neuroimage* (2011) **55**:320–328. doi:10.1016/j.neuroimage.2010.11.075

30. Carletti F, Woolley JB, Bhattacharyya S, Perez-Iglesias R, Fusar Poli P, Valmaggia L, Broome MR, Bramon E, Johns L, Giampietro V, et al. Alterations in White Matter Evident Before the Onset of Psychosis. *Schizophr Bull* (2012) **38**:1170–1179. doi:10.1093/schbul/sbs053

31. Katagiri N, Pantelis C, Nemoto T, Tsujino N, Saito J, Hori M, Yamaguchi T, Funatogawa T, Mizuno M. Longitudinal changes in striatum and sub-threshold positive symptoms in individuals with an ‘at risk mental state’ (ARMS). *Psychiatry Res - Neuroimaging* (2019) **285**:25–30. doi:10.1016/j.pscychresns.2019.01.008

32. Katagiri N, Pantelis C, Nemoto T, Tsujino N, Saito J, Hori M, Yamaguchi T, Funatogawa T, Mizuno M. Symptom recovery and relationship to structure of corpus callosum in individuals with an ‘at risk mental state.’ *Psychiatry Res - Neuroimaging* (2018) **272**:1–6. doi:10.1016/j.pscychresns.2017.11.016

33. Katagiri N, Pantelis C, Nemoto T, Zalesky A, Hori M, Shimoji K, Saito J, Ito S, Dwyer DB, Fukunaga I, et al. A longitudinal study investigating sub-threshold symptoms and white matter changes in individuals with an “at risk mental state” (ARMS). *Schizophr Res* (2015) **162**:7–13. doi:10.1016/j.schres.2015.01.002

34. Saito J, Hori M, Nemoto T, Katagiri N, Shimoji K, Ito S, Tsujino N, Yamaguchi T, Shiraga N, Aoki S, et al. Longitudinal study examining abnormal white matter integrity using a tract-specific analysis in individuals with a high risk for psychosis. *Psychiatry Clin Neurosci* (2017) **71**:530–541. doi:10.1111/pcn.12515

35. Krakauer K, Nordentoft M, Glenthøj BY, Raghava JM, Nordholm D, Randers L, Glenthøj LB, Ebdrup BH, Rostrup E. White matter maturation during 12 months in individuals at ultra-high-risk for psychosis. *Acta Psychiatr Scand* (2018) **137**:65–78. doi:10.1111/acps.12835

36. Roalf DR, de la Garza AG, Rosen A, Calkins ME, Moore TM, Quarmley M, Ruparel K, Xia CH, Rupert PE, Satterthwaite TD, et al. Alterations in white matter microstructure in individuals at persistent risk for psychosis. *Mol Psychiatry* (2019) doi:10.1038/s41380-019-0360-1

37. Gogtay N, Greenstein D, Lenane M, Clasen L, Sharp W, Gochman P, Butler P, Evans A, Rapoport J. Cortical brain development in nonpsychotic siblings of patients with childhood-onset schizophrenia. *Arch Gen Psychiatry* (2007) **64**:772–780. doi:10.1001/archpsyc.64.7.772

38. Greenstein D, Lerch J, Shaw P, Clasen L, Giedd J, Gochman P, Rapoport J, Gogtay N. Childhood onset schizophrenia: Cortical brain abnormalities as young adults. *J Child Psychol Psychiatry Allied Discip* (2006) **47**:1003–1012. doi:10.1111/j.1469-7610.2006.01658.x

39. Shaw P, Greenstein D, Lerch J, Clasen L, Lenroot R, Gogtay N, Evans A, Rapoport J, Giedd J. Intellectual ability and cortical development in children and adolescents. *Nature* (2006) **440**:676–9. doi:10.1038/nature04513

40. Mattai AA, Weisinger B, Greenstein D, Stidd R, Clasen L, Miller R, Tossell JW, Rapoport JL, Gogtay N. Normalization of cortical gray matter deficits in nonpsychotic siblings of patients with childhood-onset schizophrenia. *J Am Acad Child Adolesc Psychiatry* (2011) **50**:697–704. doi:10.1016/j.jaac.2011.03.016

41. Zalesky A, Pantelis C, Cropley V, Fornito A, Cocchi L, McAdams H, Clasen L, Greenstein D, Rapoport JL, Gogtay N. Delayed development of brain connectivity in adolescents with schizophrenia and their unaffected siblings. *JAMA Psychiatry* (2015) **72**:900–908. doi:10.1001/jamapsychiatry.2015.0226

42. Gogtay N, Hua X, Stidd R, Boyle CP, Lee S, Weisinger B, Chavez A, Giedd JN, Clasen L, Toga AW, et al. Delayed white matter growth trajectory in young nonpsychotic siblings of patients with childhood-onset schizophrenia. *Arch Gen Psychiatry* (2012) **69**:875–884. doi:10.1001/archgenpsychiatry.2011.2084

43. Greenstein D, Lenroot R, Clausen L, Chavez A, Vaituzis AC, Tran L, Gogtay N, Rapoport J. Cerebellar development in childhood onset schizophrenia and non-psychotic siblings. *Psychiatry Res - Neuroimaging* (2011) **193**:131–137. doi:10.1016/j.pscychresns.2011.02.010

44. Mattai A, Hosanagar A, Weisinger B, Greenstein D, Stidd R, Clasen L, Lalonde F, Rapoport J, Gogtay N. Hippocampal volume development in healthy siblings of childhood-onset schizophrenia patients. *Am J Psychiatry* (2011) **168**:427–435. doi:10.1176/appi.ajp.2010.10050681

45. Calvo A, Roddy DW, Coughlan H, Kelleher I, Healy C, Harley M, Clarke M, Leemans A, Frodl T, O’Hanlon E, et al. Reduced hippocampal volume in adolescents with psychotic experiences: A longitudinal population-based study. *PLoS One* (2020) **15**: doi:10.1371/journal.pone.0233670

46. Brans RGH, Van Haren NEM, Van Baal GCM, Staal WG, Schnack HG, Kahn RS, Hulshoff Pol HE. Longitudinal MRI study in schizophrenia patients and their healthy siblings. *Br J Psychiatry* (2008) **193**:422–423. doi:10.1192/bjp.bp.107.041467

47. de Wit S, Wierenga LM, Oranje B, Ziermans TB, Schothorst PF, van Engeland H, Kahn RS, Durston S. Brain development in adolescents at ultra-high risk for psychosis: Longitudinal changes related to resilience. *NeuroImage Clin* (2016) **12**:542–549. doi:10.1016/j.nicl.2016.08.013

48. Koutsouleris N, Gaser C, Bottlender R, Davatzikos C, Decker P, Jäger M, Schmitt G, Reiser M, Möller HJ, Meisenzahl EM. Use of neuroanatomical pattern regression to predict the structural brain dynamics of vulnerability and transition to psychosis. *Schizophr Res* (2010) **123**:175–187. doi:10.1016/j.schres.2010.08.032

49. Cannon TD, Chung Y, He G, Sun D, Jacobson A, Van Erp TGM, McEwen S, Addington J, Bearden CE, Cadenhead K, et al. Progressive reduction in cortical thickness as psychosis develops: A multisite longitudinal neuroimaging study of youth at elevated clinical risk. *Biol Psychiatry* (2015) **77**:147–157. doi:10.1016/j.biopsych.2014.05.023

50. Alloza C, Cox SR, Blesa Cábez M, Redmond P, Whalley HC, Ritchie SJ, Muñoz Maniega S, Valdés Hernández M del C, Tucker-Drob EM, Lawrie SM, et al. Polygenic risk score for schizophrenia and structural brain connectivity in older age: A longitudinal connectome and tractography study. *Neuroimage* (2018) **183**:884–896. doi:10.1016/j.neuroimage.2018.08.075

51. Sugranyes G, de la Serna E, Ilzarbe D, Pariente JC, Borras R, Romero S, Rosa M, Baeza I, Moreno MD, Bernardo M, et al. Brain structural trajectories in youth at familial risk for schizophrenia or bipolar disorder according to development of psychosis spectrum symptoms. *J Child Psychol Psychiatry Allied Discip* (2020) doi:10.1111/jcpp.13321
